# Exploratory Factor Analyses of the French WISC-V (WISC-V^FR^) for Five Age Groups: Analyses Based on the Standardization Sample

**DOI:** 10.1177/10731911211005170

**Published:** 2021-04-02

**Authors:** Thierry Lecerf, Gary L. Canivez

**Affiliations:** 1 University of Geneva, Geneva, Switzerland; 2 Swiss Distance Learning University, Brig, Switzerland; 3 University of Lausanne, Lausanne, Switzerland; 4 Eastern Illinois University, Charleston, IL, USA

**Keywords:** age, WISC-V^FR^, exploratory factor analyses, Schmid–Leiman (SL) procedure, structural validity, factor extraction criteria, age differentiation hypothesis

## Abstract

This study investigated the factor structure of the French Wechsler Intelligence Scale for Children–Fifth Edition with five standardization sample age groups (6-7, 8-9, 10-11, 12-13, 14-16 years) using hierarchical exploratory factor analysis followed by Schmid–Leiman procedure. The primary research questions included (a) how many French Wechsler Intelligence Scale for Children–Fifth Edition factors should be extracted and retained in each age subgroup, (b) how are subtests associated with the latent factors, (c) was there evidence for the publisher’s claim of five first-order factors and separate Visual Spatial and Fluid Reasoning factors, (d) what proportion of variance was due to general intelligence versus the first-order group ability factors following a Schmid–Leiman procedure, and (e) do results support the age differentiation hypothesis? Results suggested that four factors might be sufficient for all five age groups and results did not support the distinction between Visual Spatial and Fluid Reasoning factors. While the general factor accounted for the largest portions of variance, the four first-order factors accounted for small unique portions of variance. Results did not support the age differentiation hypothesis because the number of factors remained the same across age groups, and there was no change in the percentage of variance accounted for by the general factor across age groups.

## Public Significance Statement

The present investigation indicated that the structural validity of the French Wechsler Intelligence Scale for Children–Fifth Edition (WISC-V^FR^) with five standardization sample age groups consists of a general intelligence factor and *four* first-order primary factors. Data were not consistent with the higher-order five-factor model recommended by the publisher. The general intelligence factor accounted for the largest portion of common variance, hence supported the primary and likely exclusive interpretation of the Full Scale Intelligence Quotient (FSIQ). Results did not support the age differentiation hypothesis.

About every 10 years, intelligence tests are revised and most frequently include substantive changes. In the WISC-V^FR^ (Wechsler, 2016), three new subtests were introduced and three subtests were removed. Regarding the 12 retained subtests, several changes were introduced, and some modifications were made regarding the composite scores. These modifications resulted in questions about the internal validity. While the structural validity of the total WISC-V^FR^ standardization sample was recently evaluated by Lecerf and Canivez (2018), no independent study of the factor structure has been conducted with separate standardization sample age groups. Furthermore, based on the age differentiation theory (Garrett, 1946), and Cattell’s investment theory (1987), the extent to which relations between different broad abilities depend on age should be examined, because this theory suggested changes in the organization of intelligence with age.

The purpose of this study was to apply hierarchical exploratory factor analysis (HEFA) to five WISC-V^FR^ standardization sample age groups (6-7, 8-9, 10-11, 12-13, 14-16 years), because EFA was not reported in the WISC-V^FR^ *Interpretive Manual*, and because the reported confirmatory factor analysis (CFA) contained several psychometric concerns (Lecerf & Canivez, 2018). Complementary EFA with these five-standardization sample age groups was needed. After determining how many WISC-V^FR^ factors should be extracted and retained in each age subgroup and how the subtests are associated with the latent factors, the proportions of variance due to the second-order general intelligence factor versus the first-order group ability factors following a SL procedure (Schmid & Leiman, 1957) were estimated.

## WISC-V^FR^

The WISC-V^FR^ includes 15 subtests, which are combined to form a higher-order model consisting of a general intelligence factor with five first-order primary factors index scores. On the basis of the CHC *compendium* of cognitive abilities (Schneider & McGrew, 2018), the main theoretical goal of the WISC-V^FR^ publisher was to split the previous Perceptual Reasoning (PR) factor into two distinct factors: Visual Spatial (VS) and Fluid Reasoning (FR). The five WISC-V^FR^ first-order factors are hence consistent with the CHC *compendium* of cognitive abilities: Comprehension-Knowledge (*Gc*: VC), Visual-Spatial processing (*Gv*: VS), Fluid Reasoning (*Gf*: FR); Short-Term Working Memory (*Gwm*: WM), and Processing Speed (*Gs*: PS). In addition, although Arithmetic cross-loaded on the latent VC, FR, and WM factors, it is now considered as an indicator of FR instead of WM as in the previous WISC-IV^FR^. All other 14 subtests were associated with only one latent factor. However, this general WISC-V^FR^ structure was not supported by independent complementary EFAs and CFAs conducted with the total standardization sample (Lecerf & Canivez, 2018). The primary debates concerned the separation of VS and FR factors versus a single PR factor and the abilities assessed by the Arithmetic subtest.

The WISC-V^FR^ publisher reportedly tested this higher-order five-factor model with the five standardization sample age groups and indicated that the model fitted data best out of several competing models for all age groups. Because few CFA details were reported in the WISC-V^FR^ *Interpretive Manual* with the five sample age groups, and most importantly, because the CFAs contained several psychometric concerns, complementary EFAs with these five-standardization sample age groups was required. It was important to empirically evaluate the structural validity of the WISC-V^FR^ for the five age samples, and not only for the total sample.

## WISC-V^FR^/WISC-V Factor Structure Research

HEFA and CFA are commonly used to investigate the internal structure of subtest scores, and to provide validity evidence for the underlying latent constructs (Watkins, 2018). The factorial structure of the WISC-V^FR^ was established exclusively through CFAs. However, since many changes were introduced in the WISC-V, analyses should start with EFA and the factorial structure of the WISC-V^FR^ should not be based only on CFAs (Canivez et al., 2016). Furthermore, and as indicated by several researchers, psychometric concerns can be raised regarding the WISC-V^FR^ factorial structure and CFAs reported in the WISC-V^FR^ *Interpretive Manual* (Beaujean, 2016). These concerns also apply to the CFA conducted with the five standardization sample age groups (Canivez, Dombrowski, et al., 2018; Dombrowski, Canivez, et al., 2018). Additionally, the WISC-V^FR^ publisher determined model consistency with data solely on the basis of absolute and relative fit indexes. No information was provided regarding local model misfit and the interpretability of parameter estimates (loadings, path coefficients, etc.). With the favored WISC-V^FR^ measurement model for the total sample (labeled Model 5e), local model misfit revealed: (a) a nonstatistically significant loading of VC on Arithmetic (.02); and (b) a standardized path coefficient between *g* and FR (*Gf*) higher than 1.00, suggesting that the WISC-V^FR^ is likely overfactored. Local model misfit of the publisher’s preferred model for the total sample suggested that this model did not fit these data. There is no information regarding the local model misfit for the five standardization sample age groups. Finally, the publisher of the WISC-V^FR^ favored a five-factor higher-order model, but a higher-order model with four first-order factors exhibited equivalent goodness-of-fit indices (comparative fit index, root mean square error of approximation, Tucker–Lewis index). Therefore, there is doubt about the claim that the five first-order factors model fit best out of several competing models.

Another criticism is that the WISC-V^FR^ publisher did not decompose the subtest variance accounted for the general intelligence factor versus the five first-order group factors. Previous studies indicated that most of the total and the common variance of the WISC subtests was associated with the general intelligence factor, while small variance portions were unique to the lower group factors, except for the PS subtests (Canivez et al., 2016). This result suggested that the Wechsler scales of intelligence might be primarily measures of general intelligence. Although it is well demonstrated that the classical estimates of reliability (i.e., alpha) are biased, the publisher of the WISC-V^FR^ did not report omega-hierarchical (ω_H_) and omega-hierarchical subscale (ω_HS_) or other model based estimates such as the *H* coefficient (Hancock & Mueller, 2001), which have been shown to be more adequate (Rodriguez et al., 2016). These indices (ω_H_, ω_HS_, *H*) were estimated in the present study.

## Age-Differentiation Hypothesis

With regard to the study of human intelligence, a central topic concerns changes in the factor structure of intelligence. Several hypotheses have arisen to account for these changes: age-differentiation hypothesis, ability-differentiation hypothesis, developmental ability-differentiation hypothesis, performance-differentiation hypothesis, and developmental personality-differentiation hypothesis (Reinert, 1970). The present study focused on the age-differentiation hypothesis, which assumes that the role of the general factor becomes less important with age. According to Cattell’s investment theory, the structure of cognitive abilities becomes more differentiated with development, predicting an increase in the number and the importance of broad abilities with age. The model proposed by Ackerman (2018), the *Intelligence-as-Process, Personality, Interests, and Intelligence-as-Knowledge* (PPIK), could be considered as an “extension” of Cattell’s investment theory. The investment of *Intelligence-as-Process* leads to the development of *Intelligence-as-Knowledge*. The age-differentiation hypothesis was later extended as an age differentiation-dedifferentiation hypothesis. Over the course of development, the structure of intelligence is expected to become more differentiated in a first step, and less differentiated in a second step.

Age differentiation was mainly tested by comparing age subgroups with respect to the mean subtest correlation, and/or the first principal component and/or the factor structure. It has been suggested that the subtest correlations and the first principal component of the subtests score diminish with age, while the number of factors increase with age. Contradictory results have been reported with some studies supporting the age differentiation hypothesis (Deary et al., 1996), while others supported age dedifferentiation (Breit et al., 2020), or others age in differentiation (Escorial et al., 2003).

Some studies investigated the age differentiation hypothesis using multiple-group factor analysis (MGCFA) and results were inconsistent. Molenaar et al. (2010) and Hildebrandt et al. (2016) suggested that inconsistency is due to suboptimal methods (creation of arbitrary subgroups formed on the basis of arbitrary criteria of ability level or age) and a lack of an explicit theory of differentiation effect. These authors suggested that the age variable, which is a continuous variable, is regularly treated as a categorical one, and that this could lead to misleading results. In addition, the age categories are based on arbitrary cutoff, which are different across studies. They proposed to use moderated factor analysis (MFA) and local structural equation modeling (LSEM). However, because the first goal was to examine the factor structure of the WISC-V^FR^ with exploratory methods (EFA instead of CFA), and because raw data were unavailable, use of MFA or LSEM was not possible.

The present study assessed the age differentiation hypothesis and the factorial structure of the WISC-V^FR^ by examining whether the number of factors increased with age, and whether the proportion of variance accounted for by the general factor decreased with age. Although Lecerf and Canivez (2018) assessed the structural validity of the WISC-V^FR^ with the total standardization sample, it is possible that different structures might be observed within different age ranges. The factorial structure observed with the total sample does not guaranty that it is appropriate for each sample age group. This information is contained neither in the WISC-V^FR^ *Interpretive Manual* nor in independent studies. This investigation was necessary not only to determine the consistency of the WISC-V^FR^ structure across the developmental period but also to better understand the WISC-V^FR^ structure of the 15 subtests for each age group. The correlations between general intelligence factor and *Gf* (FRI/PRI), and between *Gf* (FRI/PRI) and *Gc* (VCI) were also examined.

The present study addressed five goals. The first was to estimate how many WISC-V^FR^ factors should be extracted and retained in each age subgroup. Incorrect specification of the correct number of factors can lead to poor score pattern reproduction and interpretation. Based on Lecerf and Canivez (2018) findings with the total sample, it was hypothesized that the factor structure of the WISC-V^FR^ for each sample age group would be better described by four factors. The second goal was to ascertain the exact nature of the constructs assessed by each subtest score by estimating the relationship between every latent factor and subtest score through EFA. The third goal was to determine if the publisher’s claim of five first-order factors and the distinction between VS and FR factors was supported. The fourth goal was to estimate the proportion of variance due to general intelligence versus the first-order group ability factors following the SL procedure. Finally, the age differentiation hypothesis was tested by examining whether the number of factors increased with age and whether the proportion of variance accounted for by the general factor decreased with age. The correlation between *Gc* (VCI) and *Gf* (FRI/PRI) should also decrease with age.

## Method

### Participants

The standardization sample raw data for the WISC-V^FR^ were requested from the publisher but access to this data set was denied. Therefore, the summary statistics for each age group (correlations and descriptive statistics) reported in the WISC-V^FR^ *Interpretive Manual* were used to conduct EFA. Five correlation matrices were used to represent five broad age subgroups. Each age group was composed of 80 to 104 children (6-7 [*n* = 201], 8-9 [*n* = 204], 10-11 [*n* = 200], 12-13 [*n* = 181], and 14-16 [*n* = 263]). The total standardization sample included 1,049 participants, and was stratified according to age, sex, six parental education levels, and five geographic regions. The total sample was matched to the French general census of the population made by the INSEE in 2010. Because summary statistics from participants who were members of the WISC-V^FR^ standardization sample age groups was used, ethics/IRB committee approval was not needed.

### Instrument

The WISC-V^FR^ is an individual test of intelligence for children and adolescents (6 to 16:11 years old). The Full Scale IQ (FISQ), which estimates the general intelligence, is based on the sum of 7 primary subtests: Block Design (BD), Similarities (SI), Vocabulary (VO), Matrix Reasoning (MR), Figure Weights (FW), Digit Span (DS), and Coding (CD). In addition to the 7 primary subtests used to estimate the FSIQ, Visual Puzzles (VP), Picture Span (PS), and Symbol Search (SS) are added for the estimation of the five primary indexes: Verbal Comprehension (VC: SI, VO), Visual Spatial (VS: BD, VP), Fluid Reasoning (FR: MR, FW), Working Memory (WM: DS, PS), and Processing Speed (PS: CD, SS). The FSIQ and the five indexes are standard scores (*M* = 100, *SD* = 15). Five ancillary index scores are also available: Quantitative Reasoning, Auditory Working Memory, Nonverbal, General ability, and Cognitive proficiency.

### Analyses

Best practices in EFA were followed as described by Watkins (2018). Principal axis exploratory factor analyses were used to analyze the combined WISC-V^FR^ standardization sample correlation matrices from the five age groups using SPSS 24 for Macintosh OSX. Principal axis EFA was selected for comparison to other WISC-V studies and because it often outperformed ML in the recovery of weak common factors. When factor extraction would not converge due to communality estimates exceeding 1.0 after maximum iterations (Heywood cases), the analyses iterations in principal axis factor extraction were limited to two in estimating final communality estimates (Gorsuch, 2015).

Multiple criteria were examined to determine the number of factors suggested for retention and included eigenvalues >1, the scree test, standard error of scree (*SE*_Scree_), Horn’s parallel analysis (HPA), and minimum average partials (MAP). The scree test is a subjective criterion so the *SE*_Scree_ as programmed by Watkins (2007) was used because it was reportedly the most accurate objective scree method. HPA and MAP were included because they are considered more accurate and less likely to overfactor (Frazier & Youngstrom, 2007), although in the presence of a strong general factor HPA tends to underfactor (Crawford et al., 2010). HPA indicates meaningful factors when eigenvalues from the WISC-V^FR^ standardization sample data were larger than eigenvalues produced by random data containing the same number of participants and factors. Random data eigenvalues for HPA were produced using the *Monte Carlo PCA for Parallel Analysis* computer program (Watkins, 2000) with 100 replications to provide stable eigenvalue estimates. Retained factors were subjected to promax (oblique) rotation (*k* = 4). Salient factor pattern coefficients were defined as those ≥.30 (Child, 2006). Factor solutions were examined for interpretability and theoretical plausibility with the empirical requirement that each factor should be marked by two or more salient pattern coefficients and no salient cross-loadings (Gorsuch, 2015). Subtest general intelligence factor loadings (first unrotated factor coefficients) were evaluated based on Kaufman’s (1994) criteria (≥.70 = good, .50 −.69 = fair, <.50 = poor).

Carroll (1993) argued that variance from the higher order factor must be extracted first to residualize the lower order factors, leaving them orthogonal to the higher order factor as cognitive ability subtest scores reflect combinations of both first-order *and* second-order factor variance. The Schmid and Leiman (1957) procedure has been recommended as the statistical method to estimate the influence of the general factor on a test from a higher order model (Gorsuch, 2015). The SL procedure is a reparameterization of a higher-order factor model, and orthogonalizes first- and second-order factors. Accordingly, first-order factors were orthogonalized by removing all variance associated with the second-order dimension using the SL procedure as programmed in the *MacOrtho* program (Watkins, 2004). This transforms “an oblique factor analysis solution containing a hierarchy of higher order factors into an orthogonal solution which not only preserves the desired interpretation characteristics of the oblique solution, but also discloses the hierarchical structuring of the variables” (Schmid & Leiman, 1957, p. 53).

The SL procedure may be constrained by proportionality and may be problematic with nonzero cross-loadings (Reise, 2012). Reise also noted two additional and more recent alternative exploratory bifactor methods that do not include proportionality constraints: analytic bifactor and target bifactor. However, the present application of the SL procedure was selected for direct comparison with results obtained by other researchers with the WISC or with other intelligence tests (Canivez, 2011; Canivez & Watkins, 2010; Dombrowski, McGill, et al., 2018; Golay & Lecerf, 2011; McGill & Dombrowski, 2018; Nelson & Canivez, 2012).

Omega-hierarchical and omega-hierarchical subscale coefficients were estimated, because McDonald’s ω_H_ provides a better estimate for the composite score (Rodriguez et al., 2016). ω_H_ is the model-based reliability/validity estimate for the hierarchical general intelligence factor independent of the variance of group factors. ω_HS_ is the model-based reliability/validity estimate of a group factor with all other group *and* general factors removed (Reise, 2012). Omega estimates (ω_H_ and ω_HS_) may be obtained from EFA SL solutions and were produced using the *Omega* program (Watkins, 2013). Omega-hierarchical coefficients should at a minimum exceed .50, but .75 would be preferred (Reise, 2012). Omega coefficients were supplemented with the *H* coefficient (Hancock & Mueller, 2001), which is a *construct reliability* coefficient that represents the correlation between a factor and an optimally weighted item composite. *H* coefficients are used to evaluate how well a set of items represents a latent variable. According to Rodriguez et al. (2016), high *H* values (>.80) suggest a well-defined latent variable.

## Results

### Factor Extraction Criteria Comparisons

Figures A1-A5 (Appendix A in online supplemental materials) illustrate HPA scree plots for the five WISC-V^FR^ age groups, while Table A1 (supplemental materials) summarized results from the multiple factor extraction criteria (eigenvalues >1, scree test, standard error of scree, HPA, MAP, theory) for suggesting the number factors to extract and retain. Table A1 showed only the publisher recommended/theory justified extraction of five factors. All other criteria across the five age groups recommended extraction of three or fewer factors. Results suggested retention of the same number of factors across the five age groups, in opposition to the age differentiation hypothesis. Because it is suggested that it is better to overextract than underextract (Wood et al., 1996), EFA began with extracting five factors to examine subtest associations based on the publisher’s suggested structure and to allow examination of the performance of smaller factors.

### Exploratory Factor Analyses: Five-Factor Extractions

Tables B1 through B5 (Appendix B in online supplemental materials) present exploratory factor analyses results extracting five factors for each of the five WISC-V^FR^ age groups. In each of the five age groups, extraction of five factors produced psychometrically inadequate results and no separate VS and FR factors emerged as all subtests from those purported factors (BD, VP, MR, FW) had salient loadings on the same factor (PR) excepting FW for ages 10 to 11 years and MR for ages 14 to 16 years.

For ages 6 to 7 years (see online supplemental Table B1), a Heywood case was produced, and the two subtests with salient factor pattern coefficients on the fifth factor included Picture Span (PS) and Cancellation (CA) which are not theoretically related, and PS cross-loaded on WM and Factor 5. For ages 8 to 9 years (see online supplemental Table B2) only one subtest (CA) had a salient factor pattern coefficient on the fifth factor rendering it inadequate. For ages 10 to 11 years (Table B3) only one subtest (CO) had a salient factor pattern coefficient on the fifth factor rendering it inadequate, and CO also cross-loaded on VC. Figure Weights had a salient factor pattern coefficient on WM and PS had no salient loading on any factor. For ages 12 to 13 years (see online supplemental Table B4), four subtests (IN, BD, VP, AR) had salient factor pattern coefficients on the fifth factor, but all four also cross-loaded on other factors more aligned with their theoretical dimensions. Furthermore, the fifth factor was composed of subtests spanning three different theoretical dimensions so made no sense. For ages 14 to 16 years (see online supplemental Table B5), only one subtest (PS) had a salient factor pattern coefficient on the fifth factor rendering it inadequate. MR had no salient factor pattern coefficient on any factor.

### Exploratory and Hierarchical Analyses

#### Ages 6 to 7 Years First-Order EFA

Table C1 (Appendix C in online supplemental materials) presents results of four factor extraction with promax rotation for 6- to 7-year-olds. The general intelligence factor loadings ranged from .290 to .775 and all were between the fair to good range (except FW, CD, and CA). PS and CA failed to exhibit salient factor pattern coefficients on any group factor. Table C1 illustrates robust alignment of VC, PR, PS, and WM subtests with theoretically consistent subtest associations. There were no subtests with salient cross-loadings. The moderate to high factor correlations presented in Table C1 imply a higher-order or hierarchical structure that required explication and the SL procedure was applied to better understand variance apportionment among general and group factors. Table C2 (online supplemental materials) presents results from three- and two-factor extractions; neither appeared theoretically viable.

#### Ages 6 to 7 Years SL Analyses: Four Group Factors

Results for the SL procedure of the higher-order factor analysis with four group factors are presented in Table 1. All subtests were properly associated (higher residual variance) with their theoretically proposed factor after removing general intelligence factor variance, except PS which had a higher residual loading and variance with PR. The general factor accounted for 66.4% of the common variance and accounted for individual subtest variability ranging 6.6% and 50.1%. Among group factors, VC accounted for an additional 11.3% of the common variance, PR for an additional 8.4% of the common variance, PS for an additional 9.6% of the common variance, and WM for an additional 4.3% of the common variance. The general and group factors combined measured 49.6% of the variance in WISC-V^FR^ scores.

**Table 1.** Sources of Variance in the French Wechsler Intelligence Scale for Children–Fifth Edition (WISC-V^FR^) for the Standardization Sample 6- to 7-Year-Olds (n = 201) According to a Schmid–Leiman Higher-Order Factor Model) with Four First-Order Factors.

WISC-V^FR^ Subtest	General		F1: Verbal Comprehension		F2: Perceptual Reasoning		F3: Processing Speed		F4: Working Memory		*h* ^2^	*u* ^2^
	*b*	*S* ^2^	*b*	*S* ^2^	*b*	*S* ^2^	*b*	*S* ^2^	*b*	*S* ^2^		
Similarities	.663	.440	**.454**	**.206**	.144	.021	−.146	.021	.003	.000	.688	.312
Vocabulary	.595	.354	**.557**	**.310**	−.035	.001	−.002	.000	−.031	.001	.666	.334
Information	.708	.501	**.354**	**.125**	.044	.002	.070	.005	.101	.010	.644	.356
Comprehension	.508	.258	**.443**	**.196**	−.083	.007	.115	.013	−.006	.000	.474	.526
Block Design	.594	.353	−.099	.010	**.406**	**.165**	.122	.015	.053	.003	.545	.455
Visual Puzzles	.634	.402	.017	.000	**.529**	**.280**	.036	.001	−.083	.007	.690	.310
Matrix Reasoning	.645	.416	−.006	.000	**.370**	**.137**	−.016	.000	.103	.011	.564	.436
Figure Weights	.403	.162	.067	.004	**.215**	**.046**	−.150	.023	.083	.007	.243	.757
Arithmetic	.697	.486	.103	.011	.139	.019	.125	.016	**.187**	**.035**	.566	.434
Digit Span	.693	.480	−.050	.003	.088	.008	−.033	.001	**.418**	**.175**	.666	.334
Picture Span	.497	.247	.094	.009	*.139*	*.019*	.062	.004	**.095**	**.009**	.288	.712
Letter–Number Sequencing	.646	.417	.162	.026	−.050	.003	.016	.000	**.319**	**.102**	.548	.452
Coding	.367	.135	−.049	.002	−.093	.009	**.533**	**.284**	.118	.014	.444	.556
Symbol Search	.480	.230	.058	.003	.086	.007	**.618**	**.382**	−.087	.008	.631	.369
Cancellation	.256	.066	.042	.002	.103	.011	**.215**	**.046**	−.054	.003	.127	.873
Total Variance		.330		.056		.042		.047		.021	.496	.504
Explained Common Variance		.664		.113		.084		.096		.043		
ω		.913		.854		.786		.624		.784		
ω_H_/ω_HS_		.814		.297		.242		.378		.109		
Relative ω		.892		.348		.308		.605		.139		
*H*		.892		.523		.442		.515		.270		
PUC		.800										

*Note*. Bold type indicates coefficients and variance estimates consistent with the theoretically proposed factor. Italic type indicates coefficients and variance estimates associated with an alternate factor (where cross-loading *b* was larger than for the theoretically assigned factor). *b* = loading of subtest on factor; *S*^2^ = variance explained; *h*^2^ = communality; *u*^2^ = uniqueness; ω_H_ = Omega-hierarchical (General Factor), ω_HS_ = Omega-hierarchical subscale (Group Factors), *H* = construct replicability coefficient, PUC = percent of uncontaminated correlations.

Table 1 also presents ω_H_ and ω_HS_ that were estimated based on the SL results. The ω_H_ coefficient for general intelligence factor (.814) was high and sufficient for scale interpretation; but, the ω_HS_ coefficients for the four group factors (VC, WM, PR, PS) were considerably lower (.109-.378). For the four group factors, unit-weighted composite scores based on these indicators would likely possess too little true score variance for clinical interpretation for the 6- to 7-year-old age group. The *H* coefficient for the general factor indicated the general factor was well defined by the 15 subtest scores, but the group factors were not adequately defined by their subtest scores (*Hs* < .70).

#### Ages 8 to 9 Years First-Order EFA

Table C3 (online supplemental materials) presents results of four factor extraction with promax rotation for 8- to 9-year-olds. The general intelligence factor loadings ranged from .206 to .745 and all were between the fair to good range (except CD, SS, and CA). All subtests exhibited salient factor pattern coefficients on a single group factor demonstrating simple structure. Table C3 illustrates robust subtest alignment of VC, PR, PS, and WM subtests with theoretically consistent subtest associations, except Arithmetic, which saliently loaded on PR. There were no subtests with salient cross–loadings. The moderate to high factor correlations presented in Table C3 imply a higher-order or hierarchical structure that required explication and the SL procedure was applied.

Table C4 (online supplemental materials) presents results from three- and two-factor extractions. In attempting to extract three factors, a Heywood case was observed. Neither the two factor nor the three factor model appeared viable due to merging of potentially meaningful constructs.

#### Ages 8-9 Years SL Analyses: Four Group Factors

Results for the SL procedure of the higher–order factor analysis with four group factors are presented in Table 2. All subtests were properly associated with their theoretically proposed factor after removing the general factor variance, except Arithmetic, which had a higher residual loading and variance with PR. The general factor accounted for 66.0% of the common variance and accounted for between 3.0% and 47.3% of individual subtest variability. Among the group factors, PR accounted for an additional 6.9% of the common variance, VC for an additional 10.2% of the common variance, WM for an additional 5.3% of the common variance, and PS accounted for an additional 11.6% of the common variance. The general and group factors combined to measure 51.2% of the variance in WISC-V^FR^ scores.

**Table 2.** Sources of Variance in the French Wechsler Intelligence Scale for Children–Fifth Edition (WISC-V^FR^) for the Standardization Sample 8- to 9-Year-Olds (n = 204) According to a Schmid–Leiman Higher-Order Factor Model) With Four First-Order Factors.

WISC-V^FR^ Subtest	General		F1: Perceptual Reasoning		F2: Verbal Comprehension		F3: Working Memory		F4 Processing Speed		*h* ^2^	*u* ^2^
	*b*	*S* ^2^	*b*	*S* ^2^	*b*	*S* ^2^	*b*	*S* ^2^	*b*	*S* ^2^		
Similarities	.647	.419	.057	.003	**.401**	**.161**	.064	.004	−.125	.016	.602	.398
Vocabulary	.619	.383	.005	.000	**.528**	**.279**	−.048	.002	.024	.001	.665	.335
Information	.679	.461	.076	.006	**.372**	**.138**	.027	.001	.051	.003	.609	.391
Comprehension	.495	.245	−.051	.003	**.449**	**.202**	−.020	.000	.075	.006	.455	.545
Block Design	.650	.423	**.395**	**.156**	−.074	.005	.019	.000	.056	.003	.587	.413
Visual Puzzles	.675	.456	**.435**	**.189**	−.012	.000	−.046	.002	.025	.001	.648	.352
Matrix Reasoning	.652	.425	**.350**	**.123**	.073	.005	.006	.000	−.105	.011	.564	.436
Figure Weights	.587	.345	**.243**	**.059**	.112	.013	.028	.001	−.038	.001	.418	.582
Arithmetic	.658	.433	.206	.042	.051	.003	.104	.011	.119	.014	.503	.497
Digit Span	.658	.433	.035	.001	−.065	.004	**.452**	**.204**	−.108	.012	.654	.346
Picture Span	.528	.279	.040	.002	.060	.004	**.190**	**.036**	.125	.016	.336	.664
Letter–Number Sequencing	.688	.473	−.032	.001	.057	.003	**.397**	**.158**	.034	.001	.636	.364
Coding	.333	.111	−.058	.003	.075	.006	.048	.002	**.463**	**.214**	.337	.663
Symbol Search	.390	.152	.037	.001	−.055	.003	.000	.000	**.718**	**.516**	.672	.328
Cancellation	.173	.030	.012	.000	.033	.001	−.069	.005	**.402**	**.162**	.198	.802
Total Variance		.338		.035		.052		.027		.059	.512	.488
Explained Common Variance		.660		.069		.102		.053		.116		
ω		.916		.825		.840		.796		.646		
ω_H_/ω_HS_		.814		.194		.285		.135		.489		
Relative ω		.889		.235		.340		.169		.757		
*H*		.895		.383		.498		.330		.605		
PUC		.800										
ω_H_/ω_HS_ with AR on PR		.811		.172		.285		.180		.489		

*Note*. Bold type indicates coefficients and variance estimates consistent with the theoretically proposed factor. Italic type indicates coefficients and variance estimates associated with an alternate factor (where cross-loading *b* was larger than for the theoretically assigned factor). *b* = loading of subtest on factor; *S*^2^ = variance explained; *h*^2^ = communality; *u*^2^ = uniqueness; ω_H_ = Omega-hierarchical (General Factor), ω_HS_ = Omega-hierarchical subscale (Group Factors), *H* = construct replicability coefficient, PUC = percent of uncontaminated correlations.

Table 2 also presents ω_H_ and ω_HS_ that were estimated based on the SL results. The ω_H_ coefficient for general intelligence was high and sufficient for scale interpretation; but, the ω_HS_ coefficients for the four group factors (PR, VC, WM, PS) were considerably lower (.135-.489). For comparison, Arithmetic was placed in the PR factor to examine effects on ω_H_ and ω_HS_ estimates. Table 3 shows minor changes in estimates with decreases in ω_H_ (*g*) and ω_HS_ (PR), but an increase in ω_HS_ for WM. Thus, for the four group factors, unit-weighted composite scores based on these indicators would likely possess too little true score variance for clinical interpretation for the 8- to 9-year-old age group. The *H* coefficient for the general factor indicated the general factor was well defined by the 15 subtest scores, but the group factors were not adequately defined by their subtest scores (*Hs* < .70).

**Table 3.** Sources of Variance in the French Wechsler Intelligence Scale for Children–Fifth Edition (WISC-V^FR^) for the Standardization Sample 10- to 11-Year-Olds (n = 200) According to a Schmid–Leiman Higher-Order Factor Model) With Four First-Order Group Factors.

WISC-V^FR^ Subtest	General		F1: Fluid Reasoning and Working Memory		F2: Verbal Comprehension		F3: Processing Speed		F4: Visual Spatial		*h* ^2^	*u* ^2^
	*b*	*S* ^2^	*b*	*S* ^2^	*b*	*S* ^2^	*b*	*S* ^2^	*b*	*S* ^2^		
Similarities	.700	.490	.051	.003	**.339**	**.115**	−.002	.000	.063	.004	.611	.389
Vocabulary	.703	.494	−.054	.003	**.523**	**.274**	−.022	.000	.026	.001	.772	.228
Information	.652	.425	.055	.003	**.386**	**.149**	−.003	.000	−.040	.002	.579	.421
Comprehension	.590	.348	.012	.000	**.382**	**.146**	.148	.022	−.085	.007	.523	.477
Block Design	.645	.416	−.022	.000	−.038	.001	.054	.003	**.586**	**.343**	.764	.236
Visual Puzzles	.668	.446	.109	.012	.080	.006	.002	.000	**.261**	**.068**	.533	.467
Matrix Reasoning	.653	.426	**.167**	**.028**	.090	.008	−.026	.001	.149	.022	.485	.515
Figure Weights	.655	.429	**.196**	**.038**	.093	.009	−.132	.017	.148	.022	.515	.485
Arithmetic	.661	.437	**.305**	**.093**	.067	.004	−.104	.011	−.015	.000	.545	.455
Digit Span	.616	.379	**.355**	**.126**	−.071	.005	.031	.001	−.030	.001	.512	.488
Picture Span	.586	.343	**.130**	**.017**	.077	.006	.212	.045	.059	.003	.415	.585
Letter–Number Sequencing	.733	.537	**.380**	**.144**	−.015	.000	.091	.008	−.074	.005	.696	.304
Coding	.439	.193	.003	.000	.038	.001	**.652**	**.425**	−.006	.000	.619	.381
Symbol Search	.361	.130	.000	.000	−.066	.004	**.632**	**.399**	.075	.006	.540	.460
Cancellation	.275	.076	−.027	.001	.122	.015	**.330**	**.109**	−.039	.002	.202	.798
Total Variance		.371		.030		.046		.062		.027	.535	.465
Explained Common Variance		.692		.056		.085		.116		.051		
ω		.927		.854		.861		.693		.771		
ω_H_/ω_HS_		.839		.114		.237		.480		.226		
Relative ω		.906		.134		.275		.693		.294		
*H*		.907		.334		.460		.604		.373		
PUC		.762										
ω_H_/ω_HS_ MR and FW on F4		.814		.136		.237		.480		.131		

*Note*. Bold type indicates coefficients and variance estimates consistent with the theoretically proposed factor. Italic type indicates coefficients and variance estimates associated with an alternate factor (where cross-loading *b* was larger than for the theoretically assigned factor). *b* = loading of subtest on factor; *S*^2^ = variance explained; *h*^2^ = communality; *u*^2^ = uniqueness; ω_H_ = Omega-hierarchical (General Factor), ω_HS_ = Omega-hierarchical subscale (Group Factors), *H* = construct replicability coefficient, PUC = percent of uncontaminated correlations; MR = Matrix Reasoning; FW = Figure Weights.

#### Ages 10 to 11 Years First-Order EFA

Table C5 (online supplemental materials) presents results of four factor extraction with promax rotation for 10- to 11-year-olds. The general intelligence factor loadings ranged from .324 to .766 and all were between the fair to good range (except SS and CA). All subtests exhibited salient factor pattern coefficients on a single group factor demonstrating simple structure (no cross-loadings). Table C5 illustrates robust subtest alignment for Factor 2 (VC) and Factor 3 (PS). Factor 1 included the two purported FR subtests (MR, FW) and four WM (AR, DS, PS, LN) subtests. Factor 4 included the two purported VS subtests. The moderate to high factor correlations presented in Table C5 imply a higher order or hierarchical structure that required explication and the SL procedure was applied.

Table C6 (online supplemental materials) presents results from three and two factor extractions. When three factors were extracted a simple structure emerged. Factor 1 included all PR and WM subtests, while Factor 2 (VC) included the four VC subtests and Factor 3 (PS) included all three PS subtests. When only two factors were extracted Factor 1 contained all VC, PR, and WM subtests, while Factor 2 contained the PS subtests.

#### Ages 10 to 11 Years SL Analyses: Four Group Factors

Results for the SL orthogonalization of the higher-order factor analysis with four group factors are presented in Table 3. All subtests were properly associated with the first-order factor extraction after removing general intelligence factor variance. The general factor accounted for 69.2% of the common variance and accounted for between 7.6% and 53.7% of individual subtest variability. Among the group factors, FR/WM accounted for an additional 5.6% of the common variance, VC for an additional 8.5% of the common variance, PS for an additional 11.6% of the common variance, and VS accounted for an additional 5.1% of the common variance. The general and group factors combined to measure 53.5% of the variance in WISC-V^FR^ scores.

Table 3 also presents ω_H_ and ω_HS_ that were estimated based on the SL results. The ω_H_ coefficient for general intelligence was high and sufficient for scale interpretation; but, the ω_HS_ coefficients for the four group factors (FR/WM, VC, PS, VS) were considerably lower (.114-.480). For contrast, Table C7 (online supplemental materials) presents statistics when subtests were assigned to traditional Wechsler factors. When MR and FW were assigned to PR, the ω_HS_ estimate for WM decreased slightly, while the ω_HS_ estimate for PR increased slightly. Thus, for the four group factors, unit-weighted composite scores based on these indicators would likely possess too little true score variance for clinical interpretation for the 10- to 11-year-old age group, regardless of which factor MR and FW were assigned. The *H* coefficient for the general factor indicated the general factor was well defined by the 15 subtest scores, but the group factors were not adequately defined by their subtest scores (*Hs* < .70).

#### Ages 12 to 13 Years First-Order EFA

Table C8 (online supplemental materials) presents results of four factor extraction with promax rotation for 12-13 year-olds. There were no salient subtest factor pattern coefficients on the fourth factor rendering it inadequate. Table C9 (online supplemental materials) presents results from three and two factor extractions. In the three factor extraction, the general intelligence factor loadings ranged from .315 to .776 and all were between the fair to good range (except CD, SS, and CA). When three factors were extracted all factors contained salient subtest factor pattern coefficients, but simple structure was not achieved. DS and LN cross-loaded on Factor 1 (VC) and Factor 2 (PR/WM). Factor 1 included the four VC subtests and also DS and LN. Factor 2 included all PR subtests (BD, VP, MR, FW) and WM subtests (AR, DS, PS, LN). Factor 3 (PS) included all three PS subtests (CD, SS, CA). When only two factors were extracted, Factor 1 contained all VC, PR, and WM subtests, while Factor 2 contained the PS subtests. BD cross-loaded on both factors. The moderate to high factor correlations presented in Table C9 imply a higher-order or hierarchical structure that required explication and the SL procedure was applied.

#### Ages 12 to 13 Years SL Analyses: Three Group Factors

Results for the SL orthogonalization of the higher-order factor analysis with three group factors are presented in Table 4. In attempting to conduct second-order EFA, a Heywood case was noted so the Gorsuch method of limiting iterations to two was applied. All subtests were properly associated (higher residual variance) with the first-order factor extraction after removing general intelligence factor variance except DS and LN which had higher residual variance with Factor 1 (VC). The general factor accounted for 64.0% of the common variance and accounted for between 8.6% and 53.4% of individual subtest variability. At the first-order level, VC accounted for an additional 12.6% of the common variance, PR/WM for an additional 9.1% of the common variance, and PS accounted for an additional 14.3% of the common variance. The general and group factors combined to measure 51.5% of the variance in WISC-V^FR^ scores.

**Table 4.** Sources of Variance in the French Wechsler Intelligence Scale for Children–Fifth Edition (WISC-V^FR^) for the Standardization Sample 12- to 13-Year-Olds (n = 181) According to a Schmid–Leiman Higher-Order Factor Model With Three First-Order Group Factors (2 Iteration Limit).

WISC-V^FR^ Subtest	General		F1: Verbal Comprehension		F2: Perceptual Reasoning and Working Memory		F3: Processing Speed		*h* ^2^	*u* ^2^
	*b*	*S* ^2^	*b*	*S* ^2^	*b*	*S* ^2^	*b*	*S* ^2^		
Similarities	.651	.424	**.435**	**.189**	.106	.011	−.005	.000	.624	.376
Vocabulary	.497	.247	**.611**	**.373**	−.071	.005	−.062	.004	.629	.371
Information	.639	.408	**.328**	**.108**	.174	.030	−.037	.001	.548	.452
Comprehension	.574	.329	**.549**	**.301**	−.039	.002	.103	.011	.643	.357
Block Design	.601	.361	−.090	.008	**.338**	**.114**	.187	.035	.519	.481
Visual Puzzles	.731	.534	−.082	.007	**.475**	**.226**	−.038	.001	.768	.232
Matrix Reasoning	.615	.378	.073	.005	**.319**	**.102**	−.047	.002	.488	.512
Figure Weights	.605	.366	.124	.015	**.299**	**.089**	−.102	.010	.481	.519
Arithmetic	.662	.438	.128	.016	**.284**	**.081**	.041	.002	.537	.463
Digit Span	.595	.354	*.330*	*.109*	**.161**	**.026**	−.077	.006	.495	.505
Picture Span	.558	.311	.108	.012	**.201**	**.040**	.163	.027	.390	.610
Letter–Number Sequencing	.602	.362	*.262*	*.069*	**.159**	**.025**	.075	.006	.462	.538
Coding	.308	.095	.009	.000	−.031	.001	**.654**	**.428**	.524	.476
Symbol Search	.385	.148	.083	.007	−.032	.001	**.652**	**.425**	.581	.419
Cancellation	.293	.086	−.103	.011	.074	.005	**.504**	**.254**	.356	.644
Total Variance		.330		.065		.047		.074	.515	.485
Explained Common Variance		.640		.126		.091		.143		
ω		.919		.869		.867		.754		
ω_H_/ω_HS_		.785		.327		.154		.550		
Relative ω		.855		.377		.178		.729		
*H*		.892		.580		.449		.646		
PUC		.648								
ω_H_/ω_HS_ DS and LN on F1		.775		.272		.193		.550		

*Note*. Bold type indicates coefficients and variance estimates consistent with the theoretically proposed factor. Italic type indicates coefficients and variance estimates associated with an alternate factor (where cross-loading *b* was larger than for the theoretically assigned factor). *b* = loading of subtest on factor; *S*^2^ = variance explained; *h*^2^ = communality; *u*^2^ = uniqueness; ω_H_ = Omega-hierarchical (General Factor); ω_HS_ = Omega-hierarchical subscale (Group Factors), *H* = construct replicability coefficient, PUC = percent of uncontaminated correlations; DS = Digit Span; LN = Letter–Number Sequencing.

Table 4 also presents ω_H_ and ω_HS_ that were estimated based on the SL results. The ω_H_ coefficient for general intelligence was high and sufficient for scale interpretation; but, the ω_HS_ coefficients for the four group factors (VC, PR/WM, PS) were considerably lower (.154-.550). For comparison, DS and LN subtests were placed in the VC factor to examine effects on ω_H_ and ω_HS_ estimates. Table 4 shows minor changes in estimates with small decreases in ω_H_ (general factor) and ω_HS_ (VC) but a slight increase in ω_HS_ for PR/WM. Thus, for the three group factors, unit-weighted composite scores based on these indicators would likely possess too little true score variance for clinical interpretation for the 12- to 13-year-old age group with the possible exception of PS. The *H* coefficient for the general factor indicated the general factor was well defined by the subtests scores but the group factors were not adequately defined by their subtest scores (*Hs* < .70).

#### Ages 14 to 16 Years First-Order EFA: Four Factor Extraction

Table C10 (online supplemental materials) presents results of four factor extraction with promax rotation. The general intelligence factor loadings ranged from .489 to .749 and all were within the fair to good range (except CA). All subtests exhibited salient factor pattern coefficients on a single group factor except MR and PS which cross-loaded on two factors so simple structure was not attained. Table C10 illustrates robust subtest alignment for Factor 1: VC; Factor 2: WM; Factor 3: PS; and Factor 4: PR (BD, VP, MR, FW). MR cross-loaded on Factor 2 and PS cross-loaded on Factor 4. The moderate to high factor correlations presented in Table C10 imply a higher order or hierarchical structure that required explication and the SL procedure was applied to better understand variance apportionment among general and group factors. Table C11 (online supplemental materials) presents results from three and two factor extractions.

#### Ages 14 to 16 Years SL Analyses: Four Group Factors

Results for the SL procedure of the higher-order factor analysis with four group factors are presented in Table 5. All subtests were properly associated (higher residual variance) with their theoretically proposed factor after removing general intelligence factor variance. The general factor accounted for 67.6% of the common variance and accounted for between 22.4% and 51.1% of individual subtest variability. Among the group factors, VC accounted for an additional 12.8% of the common variance, WM for an additional 5.6% of the common variance, PS for an additional 9.4% of the common variance, and PR accounted for an additional 4.7% of the common variance. The general and group factors combined to measure 54.8% of the variance in WISC-V^FR^ scores.

**Table 5.** Sources of Variance in the French Wechsler Intelligence Scale for Children–Fifth Edition (WISC-V^FR^) for the Standardization Sample 14- to 16-Year-Olds (n = 263) According to a Schmid–Leiman Higher-Order Factor Model) With Four First-Order Factors.

WISC-V^FR^ Subtest	General		F1: Verbal Comprehension		F2: Working Memory		F3: Processing Speed		F4: Perceptual Reasoning		*h* ^2^	*u* ^2^
	*b*	*S* ^2^	*b*	*S* ^2^	*b*	*S* ^2^	*b*	*S* ^2^	*b*	*S* ^2^		
Similarities	.583	.340	**.469**	**.220**	.004	.000	−.013	.000	.070	.005	.565	.435
Vocabulary	.463	.214	**.620**	**.384**	−.011	.000	−.059	.003	−.042	.002	.604	.396
Information	.543	.295	**.446**	**.199**	.060	.004	−.034	.001	.015	.000	.499	.501
Comprehension	.519	.269	**.499**	**.249**	−.004	.000	.123	.015	−.050	.003	.536	.464
Block Design	.618	.382	.156	.024	−.064	.004	.069	.005	**.289**	**.084**	.499	.501
Visual Puzzles	.709	.503	−.077	.006	−.028	.001	−.016	.000	**.481**	**.231**	.741	.259
Matrix Reasoning	.646	.417	.122	.015	.157	.025	−.034	.001	**.159**	**.025**	.483	.517
Figure Weights	.699	.489	.169	.029	.079	.006	.026	.001	**.208**	**.043**	.567	.433
Arithmetic	.659	.434	.110	.012	**.244**	**.060**	.001	.000	.067	.004	.510	.490
Digit Span	.715	.511	.073	.005	**.399**	**.159**	−.015	.000	−.029	.001	.677	.323
Picture Span	.611	.373	−.135	.018	**.198**	**.039**	.101	.010	.168	.028	.469	.531
Letter–Number Sequencing	.707	.500	−.007	.000	**.451**	**.203**	.037	.001	−.069	.005	.709	.291
Coding	.546	.298	.070	.005	−.038	.001	**.581**	**.338**	−.013	.000	.642	.358
Symbol Search	.557	.310	.005	.000	.017	.000	**.549**	**.301**	−.007	.000	.612	.388
Cancellation	.473	.224	−.106	.011	.078	.006	**.362**	**.131**	.049	.002	.374	.626
Total Variance		.371		.070		.031		.051		.026	.548	.452
Explained Common Variance		.676		.128		.056		.094		.047		
ω		.931		.824		.838		.771		.822		
ω_H_/ω_HS_		.836		.397		.157		.364		.126		
Relative ω		.898		.482		.187		.473		.153		
*H*		.904		.598		.354		.522		.317		
PUC		.800										

*Note*. Bold type indicates coefficients and variance estimates consistent with the theoretically proposed factor. *b* = loading of subtest on factor; *S*^2^ = variance explained; *h*^2^ = communality; *u*^2^ = uniqueness; ω_H_ = Omega-hierarchical (General Factor); ω_HS_ = Omega-hierarchical subscale (Group Factors), *H* = construct replicability coefficient, PUC = percent of uncontaminated correlations.

Also presented in Table 5 are ω_H_ and ω_HS_ coefficients that were estimated based on the SL results. The ω_H_ coefficient for general intelligence was high and sufficient for scale interpretation; however, the ω_HS_ coefficients for the four group factors (VC, WM, PS, PR) were considerably lower (.126-.397). Thus, for the four group factors unit-weighted composite scores based on these indicators would likely possess too little true score variance for clinical interpretation for the 14- to 16-year-old age group. The *H* coefficient for the general factor indicated the general factor was well defined by the 15 subtest scores, but the group factors were not adequately defined by their subtest scores (*Hs* < .70).

## Discussion

Despite several changes (subtests, composite scores), the publisher determined the internal validity of the WISC-V^FR^ exclusively on the basis of CFAs and favored a model with one second-order general factor and five first-order factors (VC, FR, VS, WM, PS). The WISC-V^FR^ publisher reported that this factorial structure was also appropriate for the five age group samples (6-7, 8-9, 10-11, 12-13, 14-16 years). However, several concerns regarding the WISC-V factor structure based on the CFAs also apply to the WISC-V^FR^ (Beaujean, 2016; Canivez & Watkins, 2016).

Consistent with Lecerf and Canivez (2018), who examined the factorial structure of the WISC-V^FR^ total standardization sample, the present data *did not* support a five-factor structure within any of the five WISC-V^FR^ age groups (online supplemental materials: Figures A1-A5, Tables B1 to B5, C1, C3, C5, C8, C10). EFA with forced extraction of five-factors indicated that either only one subtest had a salient factor pattern loading on the fifth factor (ages 8-9, 10-11, 14-16 years) or that subtests with salient factor pattern coefficients were not theoretically related (ages 6-7, 12-13 years).

For ages 6-7, 8-9, and 14-16 years, a four-factor structure similar to the WISC-IV was suggested. Results indicated that the VC subtests (SI, VO, IN, CO), the PR subtests (BD, VP, MR, FW), the PS subtests (CD, SS) and the WM subtests (AR, DS, LN) were associated with their “respective” attributes. For ages 10 to 11 years, results also suggested a four-factor structure. However, although the VC, PS, and VS subtests were associated with their respective attributes, a mixed FR/WM factor was observed (MR, FW, AR, DS, PS, LN). For ages 12 to 13 years, results suggested a three-factor structure with the VC subtests with DS and LN, the PS subtests, and a mixed PR/WM subtests (BD, VP, MR, FW, AR, DS, PS, LN).

Neither the five- nor four-factor models showed evidence for the distinction between VS and FR factors. There was no separation of Block Design and Visual Puzzles into a VS factor (VS) and MR and Figure Weights into a FR factor (FR). These four subtests combined into the former PR factor specified in earlier Wechsler scales. This finding indicated that the separation of FR and VS was unsuccessful in the WISC-V^FR^. Separate FR and VS factors were also not supported in the U.S. WISC-V (Canivez, Dombrowski, et al., 2018; Canivez et al., 2016), nor in the WISC-V^UK^ or the German WISC-V (Canivez et al., 2019; Canivez et al., 2020). Therefore, separate Visual-Spatial Index (VSI) and Fluid Reasoning Index (FRI) scores are likely misleading. If separate VSI and FRI scores are important, it is necessary to develop tasks which clearly separate the visual-spatial and the FR components. This does not reject the theoretical distinction between FR and VS, but such distinction is not provided by the WISC-V^FR^.

The Schmid and Leiman (1957) procedure (SL) applied to the four-factor EFA (or three-factor EFA with ages 12-13 years), and the examination of ω_H_ and ω_HS_ coefficients, indicated that the general factor accounted for the largest portion of WISC-V^FR^ variance. The common variance explained by the general factor ranged from 66.0% to 69.2% in the WISC-V^FR^, while Omega hierarchical (ω_H_) ranged from .785 to .839. For four of the five age groups, ω_H_ was higher than .80, suggesting that the total scores can be considered essentially unidimensional. Such unidimensionality was also supported by *H* indexes, which ranged from .892 to .907 for general intelligence factor, while all group factors had *H* indexes below the .70 criterion (Rodriguez et al., 2016). This finding was consistent with results obtained with most of the different cultural versions of the WISC-V (Canivez, Dombrowski, et al., 2018; Canivez, McGill, et al., 2018; Canivez et al., 2019; Canivez et al., 2020; Dombrowski, McGill, et al., 2018; Dombrowski et al., 2019; Fenollar-Cortés & Watkins, 2019; Watkins et al., 2018), and with other intelligence test batteries (Canivez, 2011; Canivez & Watkins, 2010; Dombrowski et al., 2009; Golay & Lecerf, 2011). This does not mean that the general intelligence factor corresponds to a single psychological attribute. The general factor may be a formative variable rather than a reflective variable, as suggested by Kan et al. (2019).

Omega hierarchical subscale coefficients (ω_HS_) were low and ranged from .109 (WM, age 6-7 years) to .550 (PS, age 12-13 years). Thus, with some exception for the CD, SS, and CA subtest scores, most common subtests variance was associated with the general factor rather than with their respective first-order factors. ω_HS_ ranged from .237 to .397 for the VC factor, from .126 to .242 for the PR factor, from .109 to .157 for the WM factor, and from .364 to .550 for the PS factor. Overall, these ω_HS_ coefficients were below the minimum threshold of .50 for reliable clinical interpretation (Reise et al., 2013). This finding suggested that clinicians should interpret with caution the five indices, if at all, because the unique contributions of the broad abilities were quite limited.

This finding supports a theoretical perspective more consistent with Carroll’s three-stratum model than with the Cattell–Horn extended Gf–Gc model. Indeed, while Horn excluded the general factor and considered it as a statistical artifact, Carroll demonstrated the importance of this factor. Likewise, Carroll suggested that subtest scores are explained first by the general factor, then by one or more broad ability, then by one or more narrow ability, and finally by unique variance. Although several broad abilities exist independently of the general factor, it appears that they are difficult to measure with appropriate level of precision. That is one reason why Canivez and Youngstrom (2019) suggested for the annulment of the arranged but unhappy marriage between Cattell–Horn’s and Carroll’s models suggested by the so-called CHC theory.

The Arithmetic subtest score was moved from WM in the previous WISC-IV to the FR factor in the WISC-V. However, EFA indicated that the AR score was more associated with WM for age groups 6 to 7 years and 14 to 16 years, while AR was associated with PR factor for age group 8 to 9 years, and with a mixture PR/WM factor for age groups 10 to 11 and 12 to 13 years. Contrary to the CFA reported in the WISC-V^FR^ *Interpretive Manual*, AR was never associated with VC.

The current study examined the influence of age and the age differentiation hypothesis on the structure of the WISC-V^FR^ by examining the number of factors retained for each age group and the percentage of variance accounted for by the general factor. According to the age differentiation hypothesis, it has been suggested that cognitive abilities tend to become more differentiated with increasing age and that the percentage of variance accounted for by the general factor decreased with age. Overall, our findings were not consistent with this hypothesis. We observed the same number of factors (four) for young children (6-7 and 8-9 years) and for adolescents (14-16 years). For ages 10 to 11 years, four factors were also found, although not exactly the same four factors; only VC and PS were observed with these four age groups. For ages 12 to 13 years, only three factors were found, rejecting the hypothesis that cognitive abilities tend to become *more* differentiated with increasing age, as reflected by the WISC-V^FR^.

Concerning the percentage of variance accounted for by the general factor, it varied from 78.5% for ages 12 to 13 years to 83.9% for ages 10 to 11 years. For adolescents (14-16 years), the percentage of variance accounted for by the general factor was slightly *higher* than for younger children, in opposition with the age differentiation hypothesis. The correlations between *Gc* (VCI) and *Gf* (FRI/PRI) were also relatively similar across age and varied from .617 (14-16 years) to .740 (10-11 years). For 6 to 7 years, this correlation was .635, while it was .617 for the 14 to 16 years. Finally, the correlation between general factor and *Gf* was perfect for all age samples. Thus, these findings did not support the age differentiation hypothesis.

In summary, the present study indicated there was no EFA evidence to support a five-factor structure within any of the five WISC-V^FR^ age groups. Results were more consistent with a four first-order factors model. Taken together, results suggested robust VC, WM, and PS factors for all age groups. SI, VO, IN, and CO estimate VC, whatever the age. DS and LN might be considered as appropriate indicators of WM, while it was not the case for the PS score. This finding suggested that the WISC-V^FR^ publisher failed to construct an adequate VS working memory subtest. CD and SS might be considered as indicators of PS, while CA was not consistently associated with these two subtests. The results of the present study indicated that the WISC-V^FR^ is overfactored when including five first-order factors, and that the higher-order model preferred by the WISC-V^FR^ publisher incorrectly concluded that the broad abilities provide useful information distinct from the general factor of intelligence. By reporting only higher-order models, the WISC-V^FR^ publisher overestimates the role of broad and specific abilities in subtest scores. This overfactoring could be due to the general factor’s variance omission, and/or due to failing to consider use of EFA to inform latent structure and forcing their preconceived five-factor model. In contrast, the present results indicated that the WISC-V^FR^ is primarily a measure of a general factor, because it accounts for substantially larger portions common and total subtest variance and supports the primary interpretation of the FSIQ. Although the FSIQ is not strictly equivalent to the general factor, the FSIQ is a good estimator of this general factor. Given the overwhelming dominance of the general factor, the present results indicated that interpretation of first-order factors is quite limited and problematic given the conflation of general and group factor variance in index scores.

### Limitations

In the present investigation, EFAs were conducted on the basis of the correlation matrices provided for the five age groups in the WISC-V^FR^ *Interpretive Manual*. Although the correlations reported rounded to two decimals, the similarity of our data with those reported in the WISC-V^FR^ *Interpretive Manual* should not lead to rejecting these findings.

EFAs cannot by themselves fully determine construct validity of the WISC-V^FR^ so studies of relations with external criteria are needed, such as incremental predictive validity (Canivez et al., 2014). Such a study could help determine if reliable achievement variance is incrementally accounted for by the WISC-V^FR^ factor index scores beyond that accounted for by the FSIQ. Diagnostic utility studies should also be examined to determine if differential patterns of WISC-V^FR^ factor index scores correctly identify individuals of differing clinical disorders (Canivez, 2013). However, given the small portions of true score variance uniquely contributed by the WISC-V^FR^ group factors, it is inconceivable that they would provide substantial value. Furthermore, these results also pertain to the standardization normative sample and may not generalize to clinical populations or independent samples of nonclinical groups.

Since many changes were introduced in the WISC-V, we examined the factor structure of the WISC-V^FR^ across the five age groups by conducting EFA and SL as a first step. The second step should be to examine age-related *invariance* using MGCFA to verify whether the subtests scores measured the same psychological constructs across age. It would be important to determine whether constructs are measured equivalently across the age, because the publisher did not provide any evidence about measurement invariance.

Based on the present results, the age differentiation hypothesis was not supported, as there was no evidence for age-related differences—either on the number of factors—or on the percentage of variance accounted for by the general factor. It would be preferable to test this hypothesis with age-related invariance of the WISC-V, but since our data were cross-sectional correlation matrices, we would be unable to assess longitudinal changes. Furthermore, because we used the correlation matrices for each age group reported in the WISC-V^FR^ *Interpretive Manual*, we would be unable to use age as a continuous variable and to use MFA (Molenaar et al., 2010) or a Local Structural Equation models (Hildebrandt et al., 2016). Therefore, our conclusion about the age differentiation hypothesis should be taken with caution. Finally, as suggested by Breit et al. (2020), investigating age differentiation effect without taking into account ability-differentiation cannot appropriately examine the changes in the intelligence structure.

## Conclusion

From a practical point of view, the present findings have several important implications for the interpretation of the WISC-V^FR^ subtests and the factor index scores across age. The higher-order model preferred by the publisher is not adequate across the five age groups, which could be quite problematic from a clinical point of view and may lead to errors in interpreting the scores. Practitioners must be aware that they are taking some risks when interpreting factor index scores because EFA did not support the separation of VS and FR factors in any of the five age groups. Furthermore, the present data suggested that the current working memory index was not appropriate, because PS was not associated with DS and LN. It is recommended that Letter–Number Sequencing be administered and to use the auditory working memory index as an indicator of the WM capacity. The present results suggested that primary interpretation of the WISC-V^FR^ should focus on the FSIQ, because the general intelligence factor accounts for the largest amount of the common variance. Factor index scores conflate general factor variance and unique group factor variance, which cannot be disentangled for individuals. The factor index scores cannot be considered to reflect only broad ability measurement; they include a strong contribution of the general intelligence factor.

## Supplemental Material

sj-pdf-1-asm-10.1177_10731911211005170 – Supplemental material for Exploratory Factor Analyses of the French WISC-V (WISC-VFR) for Five Age Groups: Analyses Based on the Standardization Sample Click here for additional data file. Supplemental material, sj-pdf-1-asm-10.1177_10731911211005170 for Exploratory Factor Analyses of the French WISC-V (WISC-VFR) for Five Age Groups: Analyses Based on the Standardization Sample by Thierry Lecerf and Gary L. Canivez in Assessment
