# Supplementary material for: Exploratory Factor Analyses of the French WISC-V (WISC-VFR) for Five Age Groups: Analyses Based on the Standardization Sample
Source: Assessment. 2021 Apr 2;29(6):1117–33. doi: 10.1177/10731911211005170 (PMC9301173; doi:10.1177/10731911211005170)
Supplement: sj-pdf-1-asm-10.1177_10731911211005170 – Supplemental material for Exploratory Factor Analyses of the French WISC-V (WISC-VFR) for Five Age Groups: Analyses Based on the Standardization Sample [file sj-pdf-1-asm-10.1177_10731911211005170.pdf]

Online Supplemental Materials Appendix A

Horn's parallel analysis (HPA; Horn, 1965) scree plots for the five French WISC–V age groups and factor extraction criteria results summary.

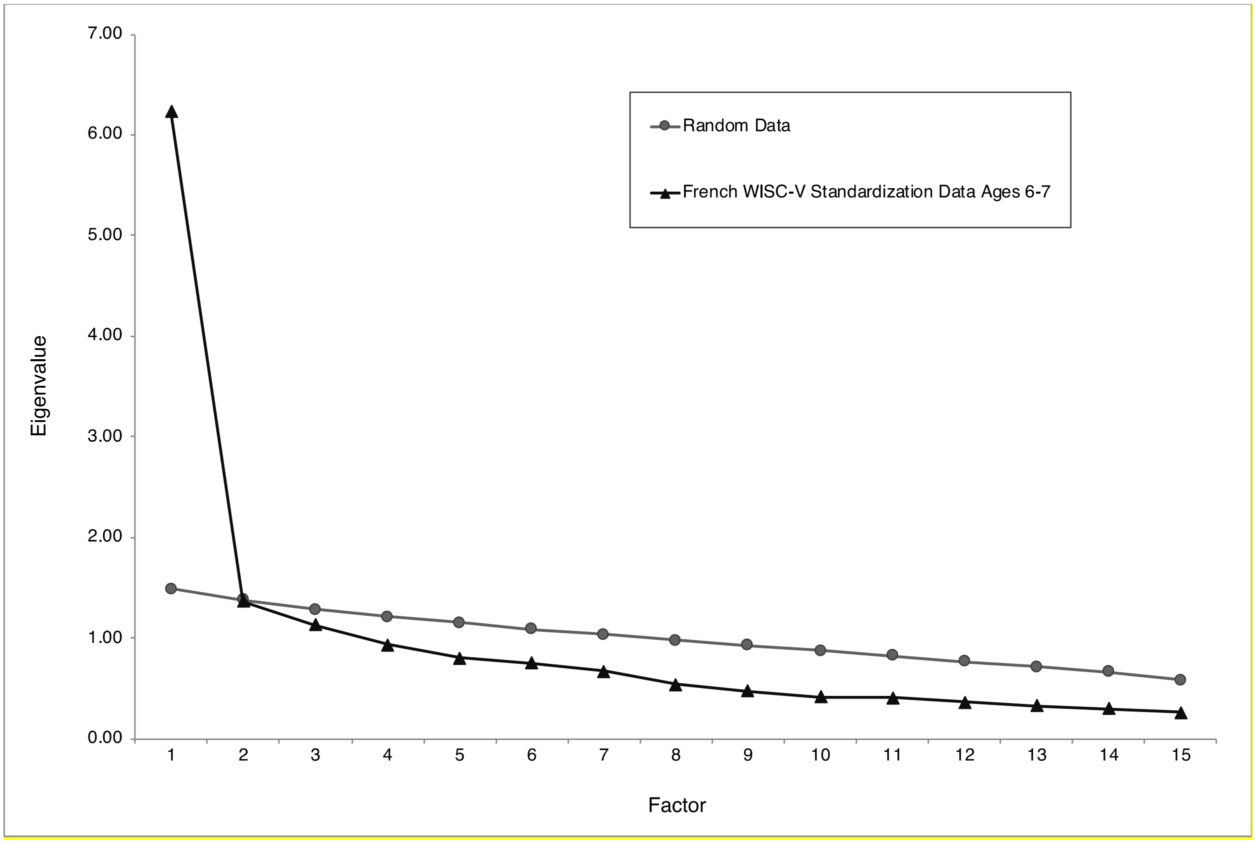

Figure A1. Scree plots for Horn's parallel analysis for French WISC–V standardization sample ages 6-7 (N = 201).

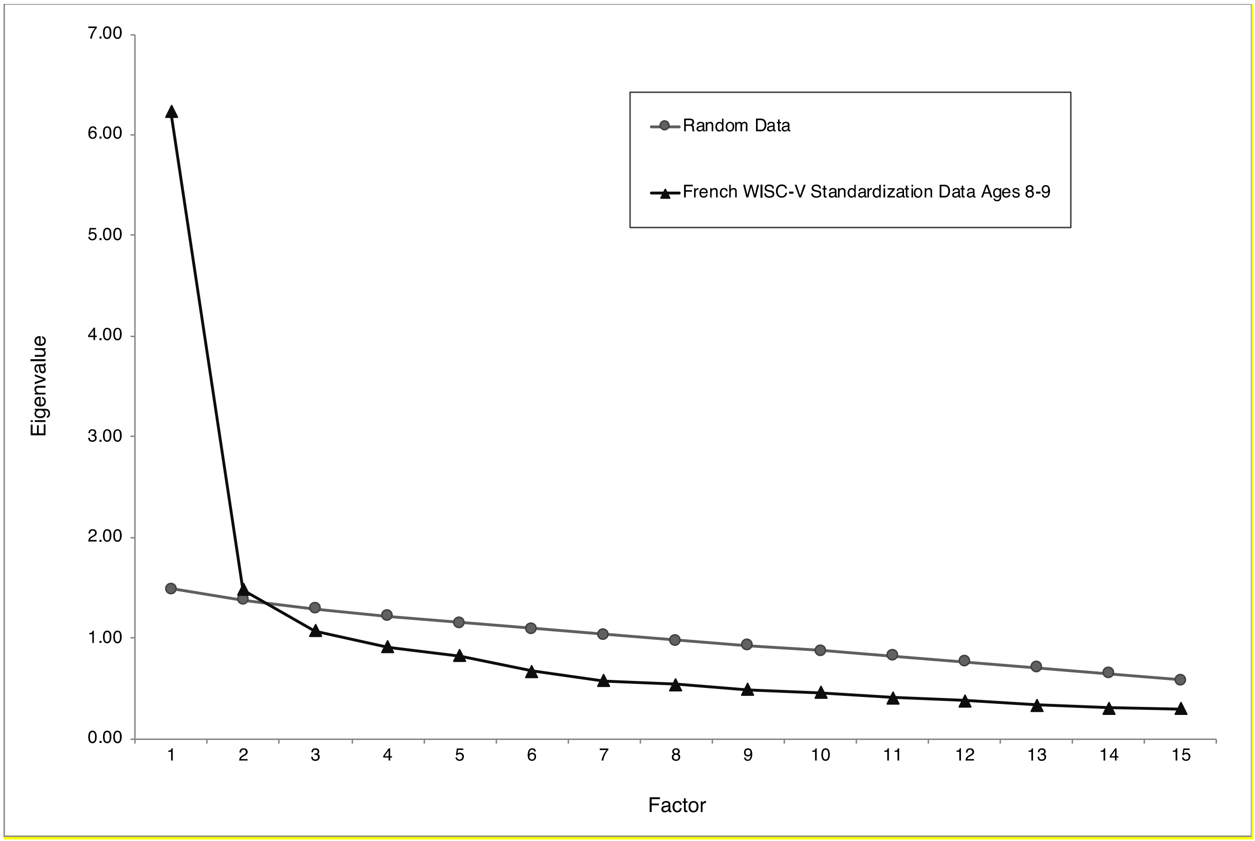

Figure A2. Scree plots for Horn's parallel analysis for French WISC–V standardization sample ages 8-9 (N = 204).

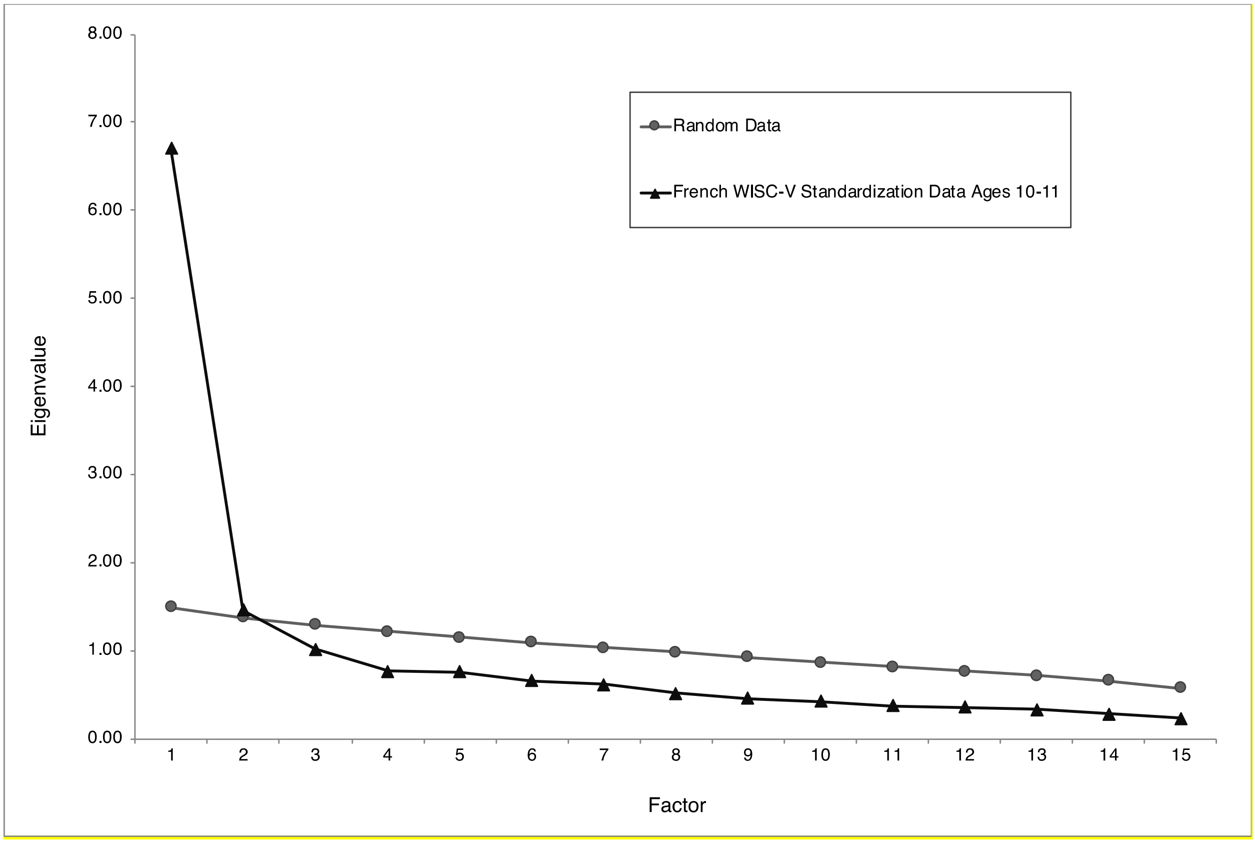

Figure A3. Scree plots for Horn's parallel analysis for French WISC–V standardization sample ages 10-11 (N = 200).

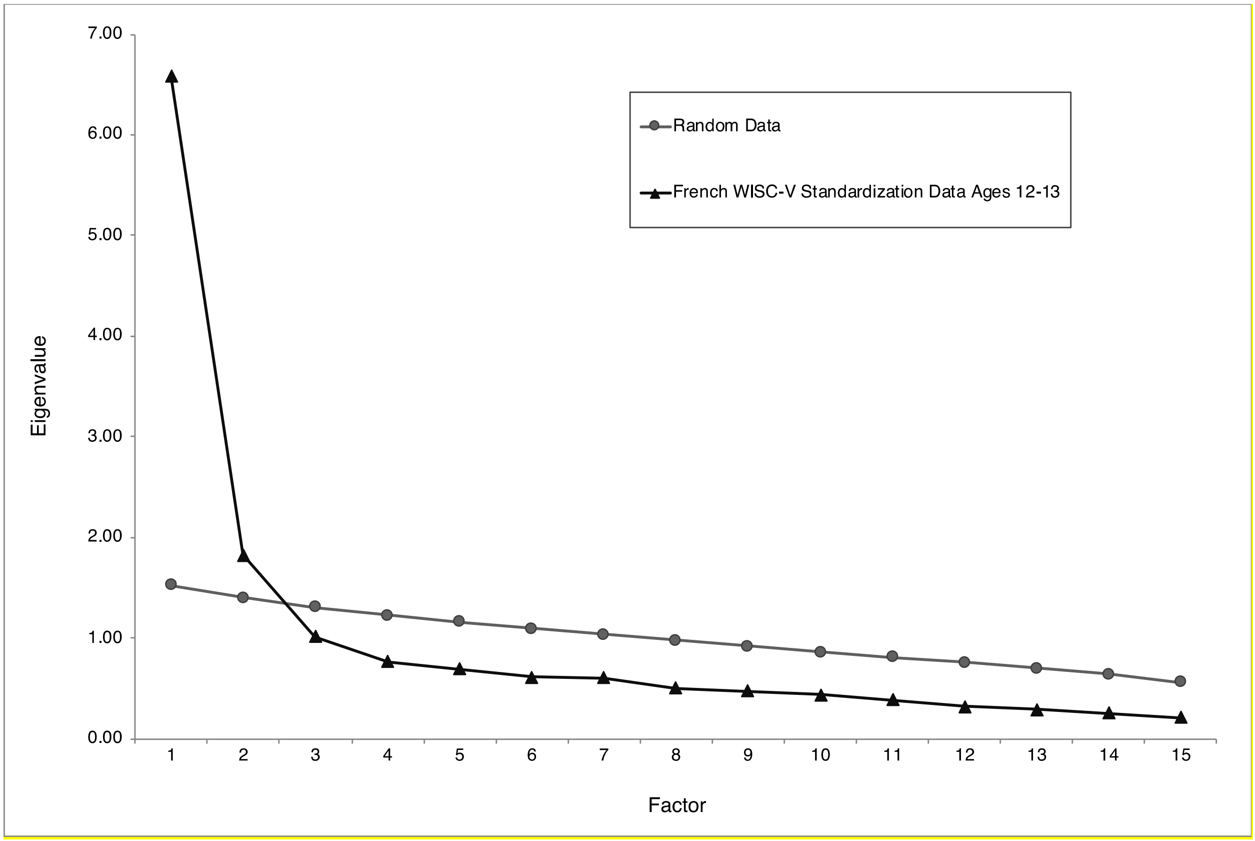

Figure A4. Scree plots for Horn's parallel analysis for French WISC–V standardization sample ages 12-13 (N = 181).

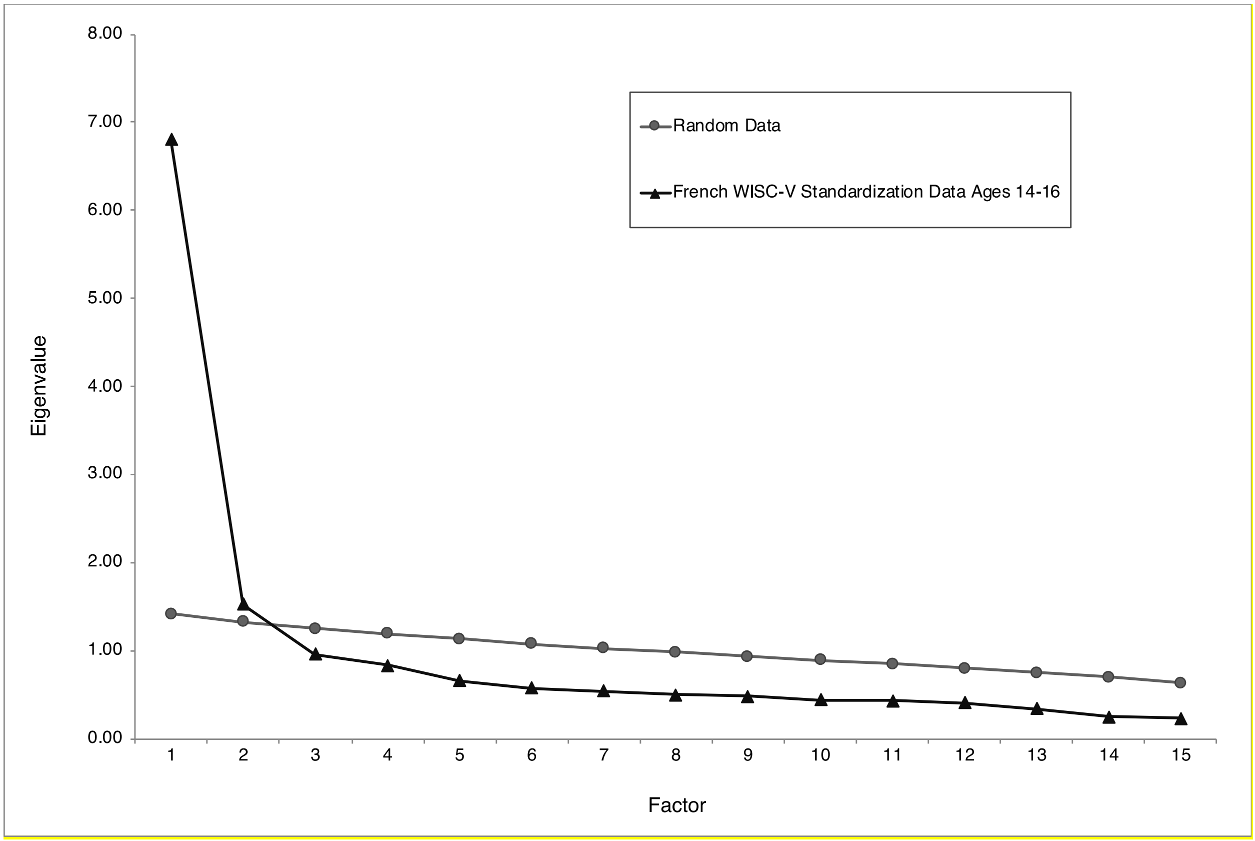

Figure A5. Scree plots for Horn's parallel analysis for French WISC–V standardization sample ages 14-16 (N = 263).

Table A1

Number of French WISC–V Factors Suggested for Extraction Across Five Different Criteria by Age Group

	WISC–V^{FR} Age Groups				
Extraction Criterion	6–7	8–9	10–11	12–13	14–16
Eigenvalue > 1	3	3	2	3	2
Scree Test (Visually Examined)	2	2	2	2	2
Standard Error of Scree (SE_{Scree})	2	4	3	3	3
Horn's Parallel Analysis (HPA)	2	2	2	2	2
Minimum Average Partials (MAP)	1	1	1	2	1
Publisher (Theory) Proposed	5	5	5	5	5

Online Supplemental Materials Appendix B

First-order exploratory factor analysis results with five extracted factors for the five French WISC–V age groups.

Table B1

French Wechsler Intelligence Scale for Children-Fifth Edition (WISC–V^{FR}) Exploratory Factor Analysis: Five Oblique Factor Solution for the Standardization Sample 6-7 Year-Olds (N = 201) with 2 iteration limit

	General	F1: Verbal Comprehension		F2: Perceptual Reasoning		F3: Processing Speed		F4: Working Memory		F5: Inadequate		
WISC–V^{FR} Subtest	*S*	*P*	*S*	*P*	*S*	*P*	*S*	*P*	*S*	*P*	*S*	h^2
SI	.736	**.712**	.797	.266	.634	-.210	.261	-.004	.567	.051	.207	.689
VO	.672	**.864**	.797	.002	.500	-.007	.332	-.093	.502	-.004	.115	.640
IN	.777	**.577**	.784	.159	.641	.117	.496	.083	.646	-.050	.124	.659
CO	.577	**.732**	.669	-.252	.370	.094	.346	.050	.456	.173	.225	.491
BD	.639	-.168	.392	**.707**	.723	.150	.484	.021	.482	.095	.334	.560
VP	.691	.031	.486	**.762**	.775	-.001	.404	-.109	.462	.205	.447	.646
MR	.690	-.014	.498	**.577**	.726	-.059	.380	.193	.571	.173	.376	.564
FW	.432	.091	.374	**.560**	.488	-.158	.161	.021	.360	-.183	-.011	.292
AR	.749	.144	.623	**.415**	.705	.239	.590	.182	.669	-.176	.053	.636
DS	.696	-.063	.537	.115	.606	-.025	.423	**.755**	.788	.085	.209	.638
PS	.542	.143	.441	.028	.457	-.011	.310	**.339**	.495	**.329**	.403	.373
LN	.671	.256	.626	-.024	.524	.062	.434	**.528**	.715	-.046	.067	.549
CD	.387	-.071	.240	-.143	.283	**.664**	.640	.140	.351	.059	.146	.426
SS	.533	.085	.369	.112	.465	**.674**	.713	-.166	.345	.150	.305	.552
CA	.293	.078	.203	.004	.250	.212	.292	-.027	.181	**.303**	.352	.180
Eigenvalue		6.23		1.37		1.13		.94		.81		
% Variance		38.58		5.78		4.06		2.48		1.74		

Factor Correlations	F1: VC	F2: PR	F3: PS	F4: WM	F5
Verbal Comprehension (VC)	–				
Perceptual Reasoning (PR)	.656	–			
Processing Speed (PS)	.450	.538	–		
Working Memory (WM)	.692	.686	.528	–	
F5	.153	.331	.190	.133	–

Note. French WISC–V Subtests: SI = Similarities, VO = Vocabulary, IN = Information, CO = Comprehension, BD = Block Design, VP = Visual Puzzles, MR = Matrix Reasoning, FW = Figure Weights, AR = Arithmetic, DS = Digit Span, PS = Picture Span, LN = Letter–Number Sequencing, CD = Coding, SS = Symbol Search, CA = Cancellation. *S* = Structure Coefficient, *P* = Pattern Coefficient, h^2 = Communality. General structure coefficients are based on the first unrotated factor coefficients (general loadings). Salient pattern coefficients presented in bold (pattern coefficient ≥ .30). Picture Span had salient factor pattern coefficients on two factors.

Table B2

French Wechsler Intelligence Scale for Children-Fifth Edition (WISC–V^{FR}) Exploratory Factor Analysis: Five Oblique Factor Solution for the Standardization Sample 8-9 Year-Olds (N = 204)

	General	F1: Perceptual Reasoning		F2: Verbal Comprehension		F3: Working Memory		F4: Processing Speed		F5: Inadequate		
WISC–V^{FR} Subtest	*S*	*P*	*S*	*P*	*S*	*P*	*S*	*P*	*S*	*P*	*S*	h^2
SI	.703	.092	.580	**.659**	.767	.124	.592	-.089	.172	-.054	.116	.611
VO	.698	.025	.545	**.863**	.814	-.118	.511	.089	.290	-.054	.157	.673
IN	.747	.118	.627	**.600**	.766	.107	.616	-.074	.270	.155	.322	.629
CO	.560	-.089	.411	**.706**	.656	-.026	.428	.071	.255	.027	.183	.439
BD	.682	**.790**	.762	-.114	.469	.052	.558	-.002	.312	.041	.286	.589
VP	.718	**.909**	.813	-.010	.528	-.125	.535	.067	.335	-.073	.219	.673
MR	.688	**.676**	.731	.138	.573	.025	.556	-.109	.186	-.047	.160	.559
FW	.625	**.475**	.625	.194	.539	.052	.512	-.025	.226	-.030	.171	.417
AR	.697	**.414**	.674	.076	.545	.213	.616	.085	.382	.062	.311	.504
DS	.652	.066	.564	-.087	.493	**.834**	.779	-.039	.240	-.103	.105	.623
PS	.554	.076	.476	.082	.445	**.392**	.557	.069	.324	.099	.279	.341
LN	.705	-.080	.565	.076	.577	**.794**	.804	.044	.347	-.001	.223	.651
CD	.393	-.080	.282	.107	.282	.002	.312	**.830**	.778	-.114	.271	.623
SS	.424	.174	.389	-.111	.233	.036	.341	**.505**	.650	.208	.485	.483
CA	.226	-.058	.183	.021	.125	-.060	.133	-.033	.332	**.868**	.823	.688
Eigenvalue		6.23		1.48		1.07		.92		.82		
% Variance		38.66		7.17		4.35		3.61		2.89		

<u>Factor Correlations</u>	F1: VC	F2: PR	F3: PS	F4: WM	F5
Verbal Comprehension (VC)	–				
Perceptual Reasoning (PR)	.682	–			
Processing Speed (PS)	.727	.684	–		
Working Memory (WM)	.391	.305	.393	–	
F5	.326	.224	.269	.466	–

Note. French WISC–V Subtests: SI = Similarities, VO = Vocabulary, IN = Information, CO = Comprehension, BD = Block Design, VP = Visual Puzzles, MR = Matrix Reasoning, FW = Figure Weights, AR = Arithmetic, DS = Digit Span, PS = Picture Span, LN = Letter–Number Sequencing, CD = Coding, SS = Symbol Search, CA = Cancellation. *S* = Structure Coefficient, *P* = Pattern Coefficient, h^2 = Communality. General structure coefficients are based on the first unrotated factor coefficients (general loadings). Salient pattern coefficients presented in bold (pattern coefficient ≥ .30).

Table B3

French Wechsler Intelligence Scale for Children-Fifth Edition (WISC–V^{FR}) Exploratory Factor Analysis: Five Oblique Factor Solution for the Standardization Sample 10-11 Year-Olds (N = 200)

	General	F1: Verbal Comprehension		F2: Working Memory		F3: Processing Speed		F4: Perceptual Reasoning		F5: Inadequate		
WISC–V^{FR} Subtest	*S*	*P*	*S*	*P*	*S*	*P*	*S*	*P*	*S*	*P*	*S*	h^2
SI	.756	**.749**	.819	.092	.635	.029	.401	-.023	.590	-.068	-.129	.679
VO	.765	**.992**	.874	-.110	.586	-.014	.371	-.031	.587	.115	.032	.783
IN	.700	**.660**	.724	.101	.589	-.010	.342	.014	.556	.174	.120	.562
CO	.661	**.624**	.649	.017	.524	.119	.409	-.008	.500	**.423**	.372	.616
BD	.652	.037	.563	-.018	.561	.111	.391	**.664**	.721	-.207	-.205	.576
VP	.723	-.081	.560	-.021	.628	-.030	.319	**.957**	.873	.132	.146	.787
MR	.676	.127	.581	.294	.644	-.035	.310	**.363**	.660	.031	.024	.493
FW	.674	.261	.647	**.375**	.664	-.116	.261	.204	.622	-.235	-.254	.574
AR	.671	.126	.578	**.620**	.713	-.125	.256	.079	.585	.014	.006	.530
DS	.630	-.081	.490	**.863**	.732	.033	.354	-.114	.491	-.083	-.074	.554
PS	.627	.135	.528	.280	.580	.236	.478	.121	.525	.050	.043	.412
LN	.756	-.059	.578	**.837**	.828	.077	.443	-.004	.617	.090	.099	.700
CD	.510	.047	.378	.015	.397	**.693**	.734	.028	.359	.156	.157	.567
SS	.433	-.080	.304	.006	.333	**.851**	.809	-.019	.279	-.100	-.087	.670
CA	.324	.143	.261	-.063	.232	**.352**	.402	.032	.233	.224	.215	.220
Eigenvalue		6.70		1.46		1.02		.77		.76		
% Variance		42.05		6.78		4.36		2.77		2.19		
<u>Factor Correlations</u>		F1: VC		F2: WM		F3: PS		F4: PR		F5		
Verbal Comprehension (VC)		–										
Working Memory (WM)		.731		–								
Processing Speed (PS)		.452		.470		–						
Perceptual Reasoning (PR)		.711		.753		.412		–				
F5		-.083		.004		.007		.008		–		

Note. French WISC–V Subtests: SI = Similarities, VO = Vocabulary, IN = Information, CO = Comprehension, BD = Block Design, VP = Visual Puzzles, MR = Matrix Reasoning, FW = Figure Weights, AR = Arithmetic, DS = Digit Span, PS = Picture Span, LN = Letter–Number Sequencing, CD = Coding, SS = Symbol Search, CA = Cancellation. *S* = Structure Coefficient, *P* = Pattern Coefficient, h^2 = Communality. General structure coefficients are based on the first unrotated factor coefficients (general loadings). Salient pattern coefficients presented in bold (pattern coefficient ≥ .30).

Table B4

French Wechsler Intelligence Scale for Children-Fifth Edition (WISC–V^{FR}) Exploratory Factor Analysis: Five Oblique Factor Solution for the Standardization Sample 12-13 Year-Olds (N = 181)

	General	F1: Verbal Comprehension		F2: Perceptual Reasoning		F3: Processing Speed		F4: Working Memory		F5: Inadequate		
WISC–V^{FR} Subtest	*S*	*P*	*S*	*P*	*S*	*P*	*S*	*P*	*S*	*P*	*S*	h^2
SI	.756	**.582**	.762	.230	.638	-.003	.255	.020	.602	.113	.416	.649
VO	.614	**.838**	.797	-.099	.413	-.057	.094	-.012	.494	.116	.301	.651
IN	.745	**.502**	.684	-.031	.570	-.016	.236	.066	.553	**.525**	.680	.715
CO	.690	**.720**	.782	.057	.529	.114	.286	.015	.567	-.013	.272	.631
BD	.645	-.026	.325	**.553**	.688	.239	.504	-.166	.390	**.304**	.589	.607
VP	.770	-.113	.436	**.575**	.797	-.017	.371	.178	.610	**.333**	.653	.728
MR	.686	.083	.499	**.881**	.788	-.089	.270	-.067	.528	-.107	.327	.641
FW	.664	.078	.504	**.464**	.660	-.121	.209	.239	.594	.078	.392	.492
AR	.745	-.047	.483	-.028	.613	.042	.356	**.615**	.730	**.434**	.630	.697
DS	.685	.250	.632	.151	.579	-.111	.180	**.474**	.708	.011	.301	.565
PS	.621	.061	.438	**.422**	.620	.168	.415	.184	.550	-.076	.270	.439
LN	.694	.100	.558	.027	.570	.061	.330	**.633**	.753	.039	.320	.581
CD	.347	.063	.138	-.085	.276	**.745**	.714	-.081	.200	.089	.236	.522
SS	.443	.035	.239	-.025	.371	**.748**	.765	.176	.395	-.157	.102	.625
CA	.313	-.101	.064	.058	.295	**.571**	.597	-.026	.191	.111	.254	.375
Eigenvalue		6.59		1.83		1.02		.77		.69		
% Variance		41.38		9.13		4.32		2.43		2.19		

Factor Correlations	F1: VC	F2: PR	F3: PS	F4: WM	F5
Verbal Comprehension (VC)	–				
Perceptual Reasoning (PR)	.579	–			
Processing Speed (PS)	.201	.449	–		
Working Memory (WM)	.661	.691	.358	–	
F5	.305	.518	.268	.347	–

Note. French WISC–V Subtests: SI = Similarities, VO = Vocabulary, IN = Information, CO = Comprehension, BD = Block Design, VP = Visual Puzzles, MR = Matrix Reasoning, FW = Figure Weights, AR = Arithmetic, DS = Digit Span, PS = Picture Span, LN = Letter–Number Sequencing, CD = Coding, SS = Symbol Search, CA = Cancellation. *S* = Structure Coefficient, *P* = Pattern Coefficient, h^2 = Communality. General structure coefficients are based on the first unrotated factor coefficients (general loadings). Salient pattern coefficients presented in bold (pattern coefficient ≥ .30). Block Design, Visual Puzzles, and Arithmetic had salient factor pattern coefficients on two factors.

Table B5

French Wechsler Intelligence Scale for Children-Fifth Edition (WISC–V^{FR}) Exploratory Factor Analysis: Five Oblique Factor Solution for the Standardization Sample 14-16 Year-Olds (N = 263)

	General	F1: Verbal Comprehension		F2: Working Memory		F3: Processing Speed		F4: Perceptual Reasoning		F5: Inadequate		
WISC–V^{FR} Subtest	*S*	*P*	*S*	*P*	*S*	*P*	*S*	*P*	*S*	*P*	*S*	h^2
SI	.669	**.729**	.758	-.060	.536	-.013	.376	.084	.556	.096	.260	.689
VO	.566	**.862**	.762	-.006	.463	-.078	.252	-.072	.419	-.059	.067	.640
IN	.625	**.698**	.717	.052	.530	-.041	.331	-.029	.486	.097	.245	.659
CO	.617	**.646**	.706	.054	.513	.177	.432	-.045	.473	-.138	.072	.491
BD	.652	.190	.543	-.118	.501	.094	.489	**.581**	.694	.030	.305	.560
VP	.698	-.132	.459	-.065	.555	-.021	.511	**.942**	.840	.106	.449	.646
MR	.677	.244	.572	.211	.618	-.049	.421	.236	.615	.223	.454	.564
FW	.743	.106	.615	.255	.669	.021	.516	**.530**	.745	-.120	.253	.292
AR	.697	.032	.556	**.596**	.723	-.013	.464	.209	.614	-.086	.271	.636
DS	.751	.032	.585	**.851**	.838	-.025	.487	-.039	.587	.018	.375	.638
PS	.619	-.044	.364	.189	.571	.132	.504	.171	.567	**.474**	.669	.373
LN	.731	.004	.531	**.832**	.820	.057	.516	-.159	.543	.149	.469	.549
CD	.602	.076	.397	-.064	.466	**.796**	.788	-.007	.501	.000	.272	.426
SS	.604	-.030	.365	.052	.497	**.767**	.786	.009	.506	-.011	.285	.552
CA	.490	-.075	.255	.051	.415	**.512**	.595	.019	.419	.207	.406	.180
Eigenvalue		6.80		1.53		.96		.83		.66		
% Variance		42.62		7.25		3.81		3.21		1.41		

Factor Correlations	F1: VC	F2: WM	F3: PS	F4: PR	F5
F1: Verbal Comprehension (VC)	–				
F2: Working Memory (WM)	.689	–			
F3: Processing Speed (PS)	.464	.606	–		
F4: Perceptual Reasoning (PR)	.661	.720	.632	–	
F5	.218	.442	.359	.434	–

Note. French WISC–V Subtests: SI = Similarities, VO = Vocabulary, IN = Information, CO = Comprehension, BD = Block Design, VP = Visual Puzzles, MR = Matrix Reasoning, FW = Figure Weights, AR = Arithmetic, DS = Digit Span, PS = Picture Span, LN = Letter–Number Sequencing, CD = Coding, SS = Symbol Search, CA = Cancellation. *S* = Structure Coefficient, *P* = Pattern Coefficient, h^2 = Communality. General structure coefficients are based on the first unrotated factor coefficients (general loadings). Salient pattern coefficients presented in bold (pattern coefficient ≥ .30). Matrix Reasoning had no salient factor pattern coefficients on any factors.

Online Supplemental Materials Appendix C

First-order exploratory factor analysis results for the five French WISC–V age groups.

Table C1

French Wechsler Intelligence Scale for Children-Fifth Edition (WISC–V^{FR}) Exploratory Factor Analysis: Four Oblique Factor Solution for the Standardization Sample 6-7 Year-Olds (N = 201)

	General	F1: Verbal Comprehension		F2: Perceptual Reasoning		F3: Processing Speed		F4: Working Memory		
WISC–V^{FR} Subtest	*S*	*P*	*S*	*P*	*S*	*P*	*S*	*P*	*S*	h^2
Similarities	.739	**.724**	.811	.243	.618	-.178	.232	.005	.568	.699
Vocabulary	.677	**.889**	.812	-.059	.467	-.002	.294	-.058	.508	.664
Information	.775	**.565**	.775	.074	.597	.085	.439	.189	.664	.645
Comprehension	.575	**.706**	.665	-.140	.370	.140	.346	-.011	.447	.461
Block Design	.638	-.158	.403	**.684**	.721	.148	.471	.098	.508	.550
Visual Puzzles	.700	.027	.505	**.892**	.830	.044	.418	-.154	.464	.700
Matrix Reasoning	.689	-.009	.509	**.623**	.732	-.020	.377	.192	.580	.555
Figure Weights	.428	.107	.370	**.363**	.441	-.182	.115	.155	.373	.236
Arithmetic	.743	.164	.611	.234	.640	.152	.504	**.349**	.688	.567
Digit Span	.701	-.079	.530	.149	.585	-.040	.385	**.779**	.802	.654
Picture Span	.534	.150	.448	.234	.481	.075	.337	.177	.467	.287
Letter–Number Sequencing	.672	.258	.617	-.084	.475	.020	.373	**.594**	.725	.557
Coding	.390	-.078	.229	-.157	.255	**.648**	.648	.220	.383	.446
Symbol Search	.544	.093	.374	.145	.469	**.752**	.780	-.162	.365	.630
Cancellation	.290	.067	.213	.174	.280	.261	.325	-.100	.187	.129
Eigenvalue		6.23		1.37		1.13		.94		
% Variance		38.69		6.09		4.34		2.76		
Promax Based Factor Correlations		F1: VC		F2: PR		F3: PS		F4: WM		
F1: Verbal Comprehension (VC)		–								
F2: Perceptual Reasoning (PR)		.635		–						
F3: Processing Speed (PS)		.397		.492		–				
F4: Working Memory (WM)		.681		.650		.491		–		

Note. *S* = Structure Coefficient, *P* = Pattern Coefficient, h^2 = Communality. General structure coefficients are based on the first unrotated factor coefficients (*general* loadings). Salient pattern coefficients presented in bold (pattern coefficient ≥ .30). Picture Span and Cancellation had no salient factor pattern coefficients.

Table C2

French Wechsler Intelligence Scale for Children-Fifth Edition (WISC–V^{FR}) Exploratory Factor Analysis: Two and Three Oblique Factor Solutions for the Standardization Sample 6-7 Year-Olds (N = 201)

	Two Oblique Factors				Three Oblique Factors				
WISC–V^{FR} Subtest	g^1	F1	F2	h^2	g^1	F1: VC	F2: PR/WM	F3: PS	h^2
SI	.741	**.890** (.813)	-.108 (.522)	.667	.740	**.736** (.797)	.240 (.628)	-.228 (.251)	.680
VO	.676	**.913** (.768)	-.205 (.442)	.611	.674	**.873** (.780)	-.102 (.484)	-.045 (.306)	.618
IN	.780	**.714** (.798)	.119 (.624)	.644	.778	**.682** (.797)	.107 (.633)	.087 (.466)	.652
CO	.572	**.683** (.627)	-.080 (.404)	.396	.576	**.741** (.658)	-.197 (.388)	.117 (.354)	.453
BD	.639	-.046 (.489)	**.755** (.723)	.523	.642	-.180 (.433)	**.783** (.734)	.138 (.492)	.564
VP	.682	.130 (.569)	**.619** (.712)	.515	.691	-.066 (.511)	**.830** (.779)	-.008 (.426)	.609
MR	.686	.210 (.593)	**.540** (.689)	.497	.692	.038 (.543)	**.736** (.750)	-.022 (.408)	.564
FW	.428	**.321** (.419)	.138 (.366)	.185	.430	.165 (.387)	**.445** (.453)	-.190 (.136)	.240
AR	.747	**.361** (.680)	**.450** (.706)	.563	.744	**.322** (.659)	**.352** (.685)	.193 (.541)	.558
DS	.684	**.359** (.630)	**.383** (.637)	.470	.681	.295 (.607)	**.385** (.642)	.090 (.444)	.466
PS	.538	.273 (.493)	**.310** (.504)	.291	.536	.230 (.476)	.288 (.502)	.094 (.363)	.288
LN	.665	**.555** (.666)	.157 (.550)	.456	.664	**.539** (.666)	.106 (.546)	.112 (.423)	.464
CD	.377	-.129 (.262)	**.552** (.460)	.220	.393	.016 (.275)	-.130 (.300)	**.748** (.682)	.476
SS	.523	-.100 (.385)	**.683** (.613)	.380	.536	.006 (.385)	.102 (.474)	**.657** (.717)	.522
CA	.291	-.036 (.219)	**.360** (.334)	.112	.291	.252 (.479)	.144 (.278)	.239 (.320)	.117
Eigenvalue		6.23	1.37			6.23	1.37	1.13	
% Variance		38.18	5.36			38.42	5.88	4.16	
Factor Correlations		F1	F2			F1	F2	F3	
	F1	–			F1	–			
	F2	.709	–		F2	.700	–		
					F3	.468	.561	–	

Note. French WISC–V Subtests: SI = Similarities, VO = Vocabulary, IN = Information, CO = Comprehension, BD = Block Design, VP = Visual Puzzles, MR = Matrix Reasoning, FW = Figure Weights, AR = Arithmetic, DS = Digit Span, PS = Picture Span, LN = Letter–Number Sequencing, CD = Coding, SS = Symbol Search, CA = Cancellation, *g* = general intelligence, VC = Verbal Comprehension, PR = Perceptual Reasoning, WM = Working Memory, PS = Processing Speed, h^2 = Communality. [1]General structure coefficients based on first unrotated factor coefficients (general loadings). Factor pattern coefficients (structure coefficients) based on principal factors extraction with promax rotation (k = 4). Salient pattern coefficients (≥ .30) presented in bold.

Table C3

French Wechsler Intelligence Scale for Children-Fifth Edition (WISC–V^{FR}) Exploratory Factor Analysis: Four Oblique Factor Solution for the Standardization Sample 8-9 Year-Olds (N = 204)

	General	F1: Perceptual Reasoning		F2: Verbal Comprehension		F3: Working Memory		F4: Processing Speed		
WISC–V^{FR} Subtest	*S*	*P*	*S*	*P*	*S*	*P*	*S*	*P*	*S*	h^2
Similarities	.704	.113	.589	**.652**	.766	.123	.590	-.143	.182	.612
Vocabulary	.697	.010	.549	**.858**	.810	-.093	.514	.028	.284	.661
Information	.745	.151	.631	**.604**	.763	.052	.599	.058	.352	.607
Comprehension	.561	-.102	.412	**.729**	.661	-.039	.425	.086	.272	.447
Block Design	.683	**.789**	.760	-.120	.472	.036	.549	.064	.387	.588
Visual Puzzles	.716	**.869**	.804	-.020	.530	-.089	.535	.029	.368	.651
Matrix Reasoning	.690	**.700**	.738	.119	.571	.012	.546	-.120	.235	.564
Figure Weights	.625	**.485**	.630	.182	.539	.053	.508	-.044	.255	.419
Arithmetic	.697	**.412**	.672	.083	.551	.199	.611	.136	.432	.503
Digit Span	.655	.070	.566	-.106	.495	**.868**	.792	-.124	.245	.645
Picture Span	.554	.080	.473	.098	.452	**.366**	.552	.143	.369	.336
Letter–Number Sequencing	.705	-.064	.565	.092	.584	**.763**	.797	.039	.371	.640
Coding	.373	-.115	.270	.122	.287	.093	.322	**.529**	.560	.331
Symbol Search	.438	.073	.373	-.090	.240	.000	.344	**.821**	.822	.680
Cancellation	.206	.024	.169	.053	.136	-.132	.120	**.460**	.432	.194
Eigenvalue		6.23		1.48		1.07		.92		
% Variance		38.60		6.52		4.17		3.23		

Promax Based Factor Correlations	F1: VC	F2: PR	F3: PS	F4: WM
F1: Verbal Comprehension (VC)	–			
F2: Perceptual Reasoning (PR)	.692	–		
F3: Processing Speed (PS)	.719	.686	–	
F4: Working Memory (WM)	.441	.341	.430	–

Note. *S* = Structure Coefficient, *P* = Pattern Coefficient, h^2 = Communality. General structure coefficients are based on the first unrotated factor coefficients (general loadings). Salient pattern coefficients presented in bold (pattern coefficient ≥ .30).

Table C4

French Wechsler Intelligence Scale for Children-Fifth Edition (WISC–V[FR]) Exploratory Factor Analysis: Two and Three Oblique Factor Solutions for the Standardization Sample 8-9 Year-Olds (N = 204)

	Two Oblique Factors				Three Oblique Factors				
WISC–V[FR] Subtest	g[1]	F1: g	F2: PS	h^2	g[1]	F1: PR/WM	F2: VC	F3: PS	h^2
SI	.705	**.837** (.736	-.212 (.185	.577	.708	.195 (.620)	**.680** (.771)	-.145 (.182)	.619
VO	.683	**.741** (.699	-.087 (.264	.495	.696	-.021 (.565)	**.801** (.790)	.013 (.280)	.624
IN	.746	**.760** (.754	-.013 (.348	.569	.748	.195 (.656)	**.604** (.763)	.049 (.350)	.606
CO	.552	**.575** (.561	-.030 (.243	.315	.563	-.128 (.434)	**.723** (.658)	.081 (.2710	.442
BD	.673	**.582** (.659	.163 (.439	.455	.684	**.853** (.749)	-.170 (.462)	.040 (.383)	.576
VP	.703	**.642** (.696	.113 (.418	.494	.711	**.824** (.764)	-.084 (.514)	.001 (.360)	.588
MR	.686	**.724** (.699	-.053 (.291	.490	.691	**.746** (.727)	.067 (.558)	-.142 (.232)	.547
FW	.629	**.642** (.636	-.014 (.291	.405	.628	**.554** (.635)	.151 (.532)	-.060 (.253)	.417
AR	.702	**.599** (.686	.184 (.468	.497	.700	**.565** (.700)	.101 (.556)	.133 (.433)	.509
DS	.633	**.638** (.639	.001 (.304	.408	.631	**.535** (.634)	.160 (.535)	-.037 (.270)	.415
PS	.556	**.462** (.541	.167 (.387	.315	.553	**.312** (.525)	.190 (.471)	.160 (.372)	.313
LN	.690	**.644** (.686	.088 (.394	.476	.686	**.396** (.651)	.295 (.612)	.087 (.375)	.471
CD	.374	.086 (.320	**.492** (.533	.290	.375	-.066 (.302)	.158 (.297)	**.539** (.563)	.330
SS	.445	-.068 (.346	**.872** (.840	.709	.440	.071 (.392)	-.094 (.243)	**.826** (.827)	.688
CA	.208	-.046 (.160	**.433** (.411	.171	.207	-.048 (.167)	.011 (.129)	**.441** (.422)	.179
Eigenvalue		6.23	1.48			6.23	1.48	1.07	
% Variance		37.99	6.45			38.32	6.51	3.99	
Factor Correlations		F1	F2			F1	F2	F3	
	F1	–			F1	–			
	F2	.475	–		F2	.725	–		
					F3	.471	.346	–	

Note. French WISC–V Subtests: SI = Similarities, VO = Vocabulary, IN = Information, CO = Comprehension, BD = Block Design, VP = Visual Puzzles, MR = Matrix Reasoning, FW = Figure Weights, AR = Arithmetic, DS = Digit Span, PS = Picture Span, LN = Letter–Number Sequencing, CD = Coding, SS = Symbol Search, CA = Cancellation, g = general intelligence, PS = Processing Speed, PR = Perceptual Reasoning, WM = Working Memory, VC = Verbal Comprehension, h^2 = Communality. [1]General structure coefficients based on first unrotated factor coefficients (general loadings). Factor pattern coefficients (structure coefficients) based on principal factors extraction with promax rotation ($k = 4$). Salient pattern coefficients (≥ .30) presented in bold.

Table C5

French Wechsler Intelligence Scale for Children-Fifth Edition (WISC–V^FR) Exploratory Factor Analysis: Four Oblique Factor Solution for the Standardization Sample 10-11 Year-Olds (N = 200)

	General	F1: Fluid Reasoning & Working Memory		F2: Verbal Comprehension		F3: Processing Speed		F4: Visual Spatial		
WISC–V^FR Subtest	*S*	*P*	*S*	*P*	*S*	*P*	*S*	*P*	*S*	h^2
Similarities	.749	.119	.647	**.618**	.770	-.002	.363	.100	.584	.610
Vocabulary	.766	-.126	.597	**.954**	.875	-.025	.356	.041	.559	.773
Information	.703	.127	.602	**.704**	.756	-.004	.344	-.063	.482	.577
Comprehension	.653	.028	.527	**.697**	.708	.171	.445	-.134	.400	.531
Block Design	.673	-.050	.587	-.069	.521	.063	.345	**.925**	.868	.759
Visual Puzzles	.696	.252	.654	.145	.599	.002	.330	**.412**	.686	.534
Matrix Reasoning	.677	**.386**	.662	.164	.589	-.030	.304	.236	.606	.486
Figure Weights	.669	**.453**	.676	.170	.589	-.153	.213	.234	.611	.516
Arithmetic	.674	**.707**	.726	.123	.577	-.120	.248	-.024	.516	.542
Digit Span	.628	**.823**	.711	-.129	.466	.036	.336	-.047	.470	.515
Picture Span	.629	**.302**	.585	.141	.534	.245	.479	.093	.489	.415
Letter–Number Sequencing	.757	**.880**	.824	-.028	.594	.105	.451	-.117	.531	.694
Coding	.515	.008	.397	.070	.406	**.755**	.786	-.010	.315	.622
Symbol Search	.425	.000	.329	-.120	.282	**.732**	.721	.119	.306	.530
Cancellation	.324	-.062	.233	.222	.306	**.382**	.431	-.061	.177	.207
Eigenvalue		6.70		1.46		1.02		.77		
% Variance		41.87		6.49		4.22		2.84		

Promax Based Factor Correlations	F1: FR/WM	F2: VC	F3: PS	F4: VS
F1: Fluid Reasoning & Working Memory (FR/WM	–			
F2: Verbal Comprehension (VC)	.740	–		
F3: Processing Speed (PS)	.456	.445	–	
F4: Visual Spatial (VS)	.713	.648	.362	–

Note. *S* = Structure Coefficient, *P* = Pattern Coefficient, h^2 = Communality. General structure coefficients are based on the first unrotated factor coefficients (general loadings). Salient pattern coefficients presented in bold (pattern coefficient ≥ .30).

Table C6

French Wechsler Intelligence Scale for Children-Fifth Edition (WISC–V^{FR}) Exploratory Factor Analysis: Two and Three Oblique Factor Solutions for the Standardization Sample 10-11 Year-Olds (N = 200)

	Two Oblique Factors				Three Oblique Factors				
WISC–V^{FR} Subtest	*g*[1]	F1: *g*	F2: PS	h^2	*g*[1]	F1: PR/WM	F2: VC	F3: PS	h^2
SI	.749	**.735** (.754)	.034 (.443)	.569	.752	.267 (.681)	**.568** (.761)	-.007 (.387)	.611
VO	.745	**.720** (.748)	.050 (.451)	.561	.769	-.008 (.639)	**.904** (.881)	-.037 (.377)	.778
IN	.699	**.674** (.701)	.048 (.423)	.493	.705	.145 (.617)	**.644** (.749)	-.004 (.365)	.570
CO	.647	**.502** (.626)	.223 (.502)	.426	.655	-.025 (.534)	**.642** (.704)	.175 (.459)	.519
BD	.641	**.625** (.644)	.034 (.382)	.416	.640	**.617** (.659)	.025 (.501)	.049 (.365)	.437
VP	.697	**.714** (.707)	-.011 (.386)	.500	.695	**.626** (.708)	.114 (.573)	-.002 (.360)	.508
MR	.681	**.718** (.695)	-.041 (.359)	.485	.679	**.631** (.698)	.111 (.561)	-.031 (.332)	.492
FW	.674	**.801** (.705)	-.173 (.273)	.517	.672	**.706** (.711)	.113 (.560)	-.159 (.242)	.527
AR	.674	**.764** (.698)	-.119 (.306)	.497	.673	**.712** (.712)	.069 (.545)	-.104 (.279)	.515
DS	.620	**.608** (.624)	.028 (.367)	.390	.624	**.753** (.675)	-.143 (.436)	.055 (.360)	.465
PS	.633	**.462** (.607)	.260 (.518)	.415	.631	**.395** (.599)	.108 (.515)	.254 (.499)	.418
LN	.746	**.684** (.742)	.104 (.485)	.558	.749	**.745** (.775)	-.044 (.562)	.125 (.473)	.612
CD	.522	-.037 (.420)	**.821** (.801)	.642	.518	-.023 (.401)	.057 (.398)	**.775** (.789)	.625
SS	.425	-.072 (.334)	**.729** (.689)	.479	.425	.053 (.341)	-.106 (.276)	**.741** (.718)	.521
CA	.326	.039 (.274)	**.423** (.445)	.199	.325	-.119 (.233)	.216 (.309)	**.391** (.432)	.208
Eigenvalue		6.70	1.46			6.70	1.46	1.02	
% Variance		41.29	6.36			41.63	6.45	3.98	
Factor Correlations		F1	F2			F1	F2	F3	
	F1	–			F1	–			
	F2	.557	–		F2	.736	–		
					F3	.493	.462	–	

Note. French WISC–V Subtests: SI = Similarities, VO = Vocabulary, IN = Information, CO = Comprehension, BD = Block Design, VP = Visual Puzzles, MR = Matrix Reasoning, FW = Figure Weights, AR = Arithmetic, DS = Digit Span, PS = Picture Span, LN = Letter–Number Sequencing, CD = Coding, SS = Symbol Search, CA = Cancellation, *g* = general intelligence, PS = Processing Speed, PR = Perceptual Reasoning, WM = Working Memory, VC = Verbal Comprehension, h^2 = Communality. [1]General structure coefficients based on first unrotated factor coefficients (general loadings). Factor pattern coefficients (structure coefficients) based on principal factors extraction with promax rotation ($k = 4$). Salient pattern coefficients (≥ .30) presented in bold.

Table C7

Sources of Variance in the French Wechsler Intelligence Scale for Children-Fifth Edition (WISC–V^{FR}) for the Standardization Sample 10-11 Year-Olds (N = 200) According to a Schmid-Leiman Higher-Order Factor Model) with Four First–Order Group Factors

	General		F1: Working Memory		F2: Verbal Comprehension		F3: Processing Speed		F4: Perceptual Reasoning			
WISC–V^{FR} Subtest	*b*	S^2	*b*	S^2	*b*	S^2	*b*	S^2	*b*	S^2	h^2	u^2
Similarities	.700	.490	.051	.003	**.339**	**.115**	-.002	.000	.063	.004	.611	.389
Vocabulary	.703	.494	-.054	.003	**.523**	**.274**	-.022	.000	.026	.001	.772	.228
Information	.652	.425	.055	.003	**.386**	**.149**	-.003	.000	-.040	.002	.579	.421
Comprehension	.590	.348	.012	.000	**.382**	**.146**	.148	.022	-.085	.007	.523	.477
Block Design	.645	.416	-.022	.000	-.038	.001	.054	.003	**.586**	**.343**	.764	.236
Visual Puzzles	.668	.446	.109	.012	.080	.006	.002	.000	**.261**	**.068**	.533	.467
Matrix Reasoning	.653	.426	*.167*	*.028*	.090	.008	-.026	.001	**.149**	**.022**	.485	.515
Figure Weights	.655	.429	*.196*	*.038*	.093	.009	-.132	.017	**.148**	**.022**	.515	.485
Arithmetic	.661	.437	**.305**	**.093**	.067	.004	-.104	.011	-.015	.000	.545	.455
Digit Span	.616	.379	**.355**	**.126**	-.071	.005	.031	.001	-.030	.001	.512	.488
Picture Span	.586	.343	**.130**	**.017**	.077	.006	.212	.045	.059	.003	.415	.585
Letter–Number Sequencing	.733	.537	**.380**	**.144**	-.015	.000	.091	.008	-.074	.005	.696	.304
Coding	.439	.193	.003	.000	.038	.001	**.652**	**.425**	-.006	.000	.619	.381
Symbol Search	.361	.130	.000	.000	-.066	.004	**.632**	**.399**	.075	.006	.540	.460
Cancellation	.275	.076	-.027	.001	.122	.015	**.330**	**.109**	-.039	.002	.202	.798
Total Variance		.330		.025		.046		.062		.030	.535	.465
Explained Common Variance		.664		.047		.085		.116		.057		
ω		.913		.808		.861		.693		.817		
ω_H / ω_{HS}		.814		.136		.237		.480		.131		
Relative ω		.892		.169		.275		.693		.160		
H		.892		.302		.460		.604		.391		
PUC		.800										
ω_H / ω_{HS} MR & FW on F1		.839		.114		.237		.480		.226		

Note. *b* = loading of subtest on factor, S^2 = variance explained, h^2 = communality, u^2 = uniqueness, ω_H = Omega hierarchical (General Factor), ω_{HS} = Omega subscale (Group Factors), MR = Matrix Reasoning, FW = Figure Weights. Bold type indicates coefficients and variance estimates consistent with the theoretically proposed factor. Italic type indicates coefficients and variance estimates associated with an alternate factor (where cross-loading *b* was larger than for the theoretically assigned factor).

Table C8

French Wechsler Intelligence Scale for Children-Fifth Edition (WISC–V^{FR}) Exploratory Factor Analysis: Four Oblique Factor Solution for the Standardization Sample 12-13 Year-Olds (N = 181)

	General	F1: Perceptual Reasoning & Working Memory		F2: Verbal Comprehension		F3: Processing Speed		F4: Inadequate		
WISC–V^{FR} Subtest	*S*	*P*	*S*	*P*	*S*	*P*	*S*	*P*	*S*	h^2
Similarities	.757	.239	.667	**.612**	.781	-.002	.258	.010	.187	.639
Vocabulary	.616	-.165	.459	**.929**	.797	-.052	.094	-.017	.193	.657
Information	.742	.278	.684	**.623**	.757	-.009	.268	-.236	-.053	.672
Comprehension	.690	-.034	.547	**.738**	.767	.111	.275	.105	.285	.609
Block Design	.646	**.653**	.704	-.059	.405	.242	.532	-.211	-.150	.596
Visual Puzzles	.774	**.948**	.853	-.105	.531	-.024	.396	-.089	-.013	.744
Matrix Reasoning	.672	**.711**	.698	.010	.516	-.065	.275	.098	.176	.501
Figure Weights	.666	**.680**	.681	.073	.545	-.131	.209	.116	.205	.499
Arithmetic	.729	**.591**	.728	.157	.589	.051	.368	.024	.127	.545
Digit Span	.686	**.435**	.629	.318	.658	-.122	.165	.262	.385	.564
Picture Span	.622	**.497**	.609	.020	.458	.160	.403	.208	.269	.433
Letter–Number Sequencing	.690	**.432**	.638	.216	.599	.053	.314	.276	.376	.525
Coding	.348	-.108	.295	.074	.172	**.751**	.717	-.032	-.011	.520
Symbol Search	.446	-.038	.364	.025	.247	**.755**	.748	.275	.291	.637
Cancellation	.314	.112	.311	-.100	.108	**.578**	.606	-.038	-.040	.376
Eigenvalue		6.59		1.83		1.02		.77		
% Variance		41.16		9.13		4.24		2.24		

Promax Based Factor Correlations	F1: VC	F2: PR	F3: PS	F4
F1: Perceptual Reasoning/Working Memory (PR/WM)	–			
F2: Verbal Comprehension (VC)	.700	–		
F3: Processing Speed (PS)	.471	.241	–	
F4	.107	.246	.019	–

Note. *S* = Structure Coefficient, *P* = Pattern Coefficient, h^2 = Communality. General structure coefficients are based on the first unrotated factor coefficients (general loadings). Salient pattern coefficients presented in bold (pattern coefficient ≥ .30). Factor 4 had no salient subtest factor pattern coefficients.

Table C9

French Wechsler Intelligence Scale for Children-Fifth Edition (WISC–V^{FR}) Exploratory Factor Analysis: Two and Three Oblique Factor Solutions for the Standardization Sample 12-13 Year-Olds (N = 181)

	Two Oblique Factors				Three Oblique Factors				
WISC–V^{FR} Subtest	g^1	F1: g	F2: PS	h^2	g^1	F1: VC	F2: PR/ WM	F3: PS	h^2
SI	.760	**.828** (.785)	-.086 (.325)	.622	.759	**.637** (.784)	.216 (.651)	-.006 (.268)	.639
VO	.603	**.790** (.655)	-.273 (.120)	.485	.615	**.896** (.779)	-.143 (.440)	-.070 (.105)	.628
IN	.734	**.781** (.754)	-.054 (.334)	.571	.731	**.481** (.712)	.353 (.663)	-.042 (.255)	.566
CO	.681	**.734** (.702)	-.064 (.300)	.496	.693	**.804** (.781)	-.080 (.528)	.117 (.296)	.621
BD	.637	**.369** (.581)	**.426** (.609)	.474	.640	-.132 (.396)	**.685** (.694)	.213 (.501)	.530
VP	.758	**.620** (.734)	.231 (.539)	.580	.776	-.120 (.530)	**.964** (.861)	-.043 (.380)	.750
MR	.671	**.617** (.666)	.099 (.405)	.451	.673	.107 (.537)	**.646** (.695)	-.053 (.282)	.492
FW	.666	**.672** (.675)	.007 (.341)	.456	.666	.181 (.567)	**.607** (.676)	-.116 (.220)	.488
AR	.732	**.638** (.719)	.162 (.479)	.537	.731	.188 (.597)	**.577** (.728)	.047 (.370)	.549
DS	.686	**.771** (.714)	-.114 (.269)	.520	.682	**.483** (.683)	**.326** (.616)	-.088 (.196)	.513
PS	.623	**.460** (.592)	.266 (.494)	.404	.620	.159 (.489)	**.407** (.604)	.185 (.421)	.401
LN	.689	**.642** (.686)	.088 (.407)	.476	.685	**.384** (.629)	**.323** (.627)	.085 (.341)	.473
CD	.346	-.119 (.239)	**.721** (.662)	.449	.348	.013 (.170)	-.063 (.297)	**.743** (.717)	.516
SS	.436	-.002 (.337)	**.684** (.683)	.466	.441	.122 (.278)	-.065 (.370)	**.741** (.743)	.561
CA	.317	-.122 (.216)	**.681** (.620)	.396	.315	-.151 (.108)	.151 (.318)	**.573** (.604)	.378
Eigenvalue		6.59	1.83			6.59	1.83	1.02	
% Variance		40.65	8.56			40.97	8.94	4.11	
Factor Correlations		F1	F2			F1	F2	F3	
	F1	–			F1	–			
	F2	.497	–		F2	.687	–		
					F3	.270	.473	–	

Note. WISC–V^{FR} Subtests: SI = Similarities, VO = Vocabulary, IN = Information, CO = Comprehension, BD = Block Design, VP = Visual Puzzles, MR = Matrix Reasoning, FW = Figure Weights, AR = Arithmetic, DS = Digit Span, PS = Picture Span, LN = Letter–Number Sequencing, CD = Coding, SS = Symbol Search, CA = Cancellation, g = general intelligence, PS = Processing Speed, PR = Perceptual Reasoning, WM = Working Memory, h^2 = Communality. [1]General structure coefficients based on first unrotated factor coefficients (general loadings). Factor pattern coefficients (structure coefficients) based on principal factors extraction with promax rotation (k = 4). Salient pattern coefficients (≥ .30) presented in bold.

Table C10

French Wechsler Intelligence Scale for Children-Fifth Edition (WISC–V^{FR}) Exploratory Factor Analysis: Four Oblique Factor Solution for the Standardization Sample 14-16 Year-Olds (N = 263)

	General	F1: Verbal Comprehension		F2: Working Memory		F3: Processing Speed		F4: Perceptual Reasoning		
WISC–V^{FR} Subtest	*S*	*P*	*S*	*P*	*S*	*P*	*S*	*P*	*S*	h^2
Similarities	.668	**.661**	.746	.009	.527	-.018	.373	.141	.544	.568
Vocabulary	.568	**.873**	.769	-.023	.427	-.083	.239	-.085	.384	.609
Information	.624	**.628**	.705	.122	.521	-.047	.328	.031	.478	.506
Comprehension	.617	**.702**	.711	-.009	.476	.172	.416	-.101	.436	.523
Block Design	.653	.219	.540	-.130	.498	.097	.489	**.586**	.688	.505
Visual Puzzles	.700	-.108	.449	-.056	.573	-.022	.519	**.976**	.855	.742
Matrix Reasoning	.677	.172	.555	**.319**	.636	-.048	.430	**.323**	.631	.481
Figure Weights	.738	.238	.617	.160	.643	.036	.509	**.422**	.708	.566
Arithmetic	.694	.155	.558	**.496**	.695	.001	.460	.135	.593	.511
Digit Span	.749	.103	.582	**.812**	.824	-.021	.486	-.058	.584	.685
Picture Span	.610	-.190	.343	**.403**	.615	.141	.520	**.340**	.607	.462
Letter–Number Sequencing	.733	-.010	.519	**.917**	.841	.052	.520	-.139	.556	.716
Coding	.603	.099	.396	-.077	.465	**.812**	.792	-.027	.496	.633
Symbol Search	.604	.007	.363	.034	.498	**.766**	.781	-.014	.504	.611
Cancellation	.489	-.149	.241	.159	.445	**.506**	.600	.099	.446	.383
Eigenvalue		6.80		1.53		.96		.83		
% Variance		42.51		7.18		3.76		3.23		
Promax Based Factor Correlations		F1: VC		F2: WM		F3: PS		F4: PR		
F1: Verbal Comprehension (VC)		–								
F2: Working Memory (WM)		.645		–						
F3: Processing Speed (PS)		.447		.613		–				
F4: Perceptual Reasoning (PR)		.617		.729		.639		–		

Note. *S* = Structure Coefficient, *P* = Pattern Coefficient, h^2 = Communality. General structure coefficients are based on the first unrotated factor coefficients (general loadings). Salient pattern coefficients presented in bold (pattern coefficient ≥ .30). Matrix Reasoning had salient factor pattern coefficients on F2 and F4.

Table C11

French Wechsler Intelligence Scale for Children-Fifth Edition (WISC–V^{FR}) Exploratory Factor Analysis: Two and Three Oblique Factor Solutions for the Standardization Sample 14-16 Year-Olds (N = 263)

	Two Oblique Factors				Three Oblique Factors				
WISC–V^{FR} Subtest	*g*[1]	F1	F2	h^2	*g*[1]	F1: PR/WM	F2: VC	F3: PS	h^2
SI	.672	-.019 (.501)	**.761** (.748)	.559	.670	.126 (.575)	**.659** (.746)	.004 (.380)	.565
VO	.570	-.296 (.340)	**.931** (.729)	.578	.570	-.079 (.442)	**.864** (.768)	-.096 (.236)	.606
IN	.629	-.056 (.457)	**.750** (.712)	.509	.626	.166 (.545)	**.617** (.704)	-.058 (.326)	.506
CO	.616	.004 (.465)	**.674** (.677)	.458	.620	-.092 (.491)	**.703** (.712)	.161 (.413)	.522
BD	.648	**.405** (.610)	***.301*** (.578)	.421	.646	**.312** (.605)	.234 (.535)	.203 (.514)	.419
VP	.676	**.600** (.691)	.132 (.543)	.486	.674	**.576** (.687)	-.007 (.460)	.175 (.554)	.489
MR	.679	**.362** (.621)	***.379*** (.626)	.462	.680	**.605** (.689)	.157 (.552)	-.035 (.437)	.489
FW	.741	**.407** (.681)	**.401** (.679)	.550	.739	**.489** (.716)	.243 (.616)	.094 (.527)	.550
AR	.695	***.392*** (.642)	***.366*** (.634)	.483	.697	**.647** (.712)	.130 (.552)	-.034 (.453)	.517
DS	.738	**.430** (.685)	***.374*** (.668)	.544	.743	**.768** (.775)	.089 (.573)	-.081 (.469)	.609
PS	.611	**.685** (.667)	-.027 (.442)	.445	.612	**.699** (.663)	-.204 (.338)	.155 (.528)	.476
LN	.715	**.513** (.694)	.264 (.615)	.518	.720	**.763** (.758)	.003 (.515)	-.011 (.497)	.575
CD	.588	**.713** (.658)	-.080 (.407)	.436	.605	-.095 (.506)	.099 (.392)	**.805** (.785)	.623
SS	.594	**.775** (.681)	-.137 (.392)	.473	.604	.028 (.531)	.008 (.360)	**.750** (.772)	.597
CA	.491	**.752** (.597)	-.227 (.287)	.384	.491	.252 (.479)	-.159 (.236)	**.503** (.600)	.385
Eigenvalue		6.80	1.53			6.80	1.53	.96	
% Variance		41.95	6.77			42.22	7.10	3.51	
Factor Correlations		F1	F2			F1	F2	F3	
	F1	–			F1	–			
	F2	.684	–		F2	.677	–		
					F3	.664	.444	–	

Note. French WISC–V Subtests: SI = Similarities, VO = Vocabulary, IN = Information, CO = Comprehension, BD = Block Design, VP = Visual Puzzles, MR = Matrix Reasoning, FW = Figure Weights, AR = Arithmetic, DS = Digit Span, PS = Picture Span, LN = Letter–Number Sequencing, CD = Coding, SS = Symbol Search, CA = Cancellation, *g* = general intelligence, PS = Processing Speed, PR = Perceptual Reasoning, WM = Working Memory, VC = Verbal Comprehension, h^2 = Communality. [1]General structure coefficients based on first unrotated factor coefficients (general loadings). Factor pattern coefficients (structure coefficients) based on principal factors extraction with promax rotation ($k = 4$). Salient pattern coefficients (≥ .30) presented in bold.
